# Death receptor 6 contributes to autoimmunity in lupus-prone mice

**DOI:** 10.1038/ncomms13957

**Published:** 2017-01-03

**Authors:** Daisuke Fujikura, Masahiro Ikesue, Tsutomu Endo, Satoko Chiba, Hideaki Higashi, Toshimitsu Uede

**Affiliations:** 1 Division of Infection and Immunity, Hokkaido University Research Center for Zoonosis Control, North-20, West-10, Kita-ku, Sapporo 001-0020, Japan; 2 Division of Molecular Immunology, Hokkaido University Institute for Genetic Medicine, North-15, West-7, Kita-ku, Sapporo 060-0815, Japan; 3 Division of Bioresources, Hokkaido University Research Center for Zoonosis Control, North-20, West-10, Kita-ku, Sapporo 001-0020, Japan

## Abstract

Expansion of autoreactive follicular helper T (Tfh) cells is tightly restricted to prevent induction of autoantibody-dependent immunological diseases, such as systemic lupus erythematosus (SLE). Here we show expression of an orphan immune regulator, death receptor 6 (DR6/TNFRSF21), on a population of Tfh cells that are highly expanded in lupus-like disease progression in mice. Genome-wide screening reveals an interaction between syndecan-1 and DR6 resulting in immunosuppressive functions. Importantly, syndecan-1 is expressed specifically on autoreactive germinal centre (GC) B cells that are critical for maintenance of Tfh cells. Syndecan-1 expression level on GC B cells is associated with Tfh cell expansion and disease progression in lupus-prone mouse strains. In addition, Tfh cell suppression by DR6-specific monoclonal antibody delays disease progression in lupus-prone mice. These findings suggest that the DR6/syndecan-1 axis regulates aberrant GC reactions and could be a therapeutic target for autoimmune diseases such as SLE.

## Abstract (web summary)

Germinal centre (GC) reactions are driven by T follicular helper (Tfh) cells and their dysregulation can cause autoimmune disease. Here the authors show that the orphan receptor DR6 is a Tfh cell marker that binds syndecan-1 on GC B cells driving autoimmunity in lupus-prone mice.

Systemic lupus erythematosus (SLE) is a chronic inflammatory disease resulting from autoantibody recognition of self-antigens, with autoantibody production dependent on activation of autoreactive T and B cells^1^. Although autoreactive T and B cells can be detected in healthy wild-type mice^2^,^3^, the expansion and activation of these cells are tightly controlled by tolerance mechanisms. Defects in genes associated with apoptotic cell clearance cause systemic autoimmune disease in familial SLE patients and C57BL/6 (B6) mice^4^,^5^,^6^.

Normally, the activation of autoreactive lymphocytes should be regulated at the stage of initial T/B cell interactions^7^,^8^,^9^. The activation and differentiation of peripheral T and B cells requires multiple steps^10^. Antigen-primed CD4^+^ T cells migrate from the T cell zone to the B cell follicles after expressing CXCR5, which is a chemokine receptor^11^. In the lymphoid structure termed the germinal centre (GC), located on the border between the T and B cell zones, the primed CD4^+^ T cells differentiate into follicular helper T (Tfh) cells and promote B cell maturation, such as proliferation, somatic hyper maturation and immunoglobulin class switching, through its production of cytokines such as interleukin (IL)-4 and IL-21. Tfh cells express the chemokine receptor CXCR4 to migrate from the original follicle to a neighboring follicle and induce new GC formation. In these sequential steps, reciprocal signals by antigen-specific GC B cells are important for complete Tfh cell differentiation and maintenance. In promoting complete Tfh cell differentiation, the GC B cells activate T cell receptor (TCR) signalling through antigen presentation. The expression of costimulatory ligands such as inducible T cell co-stimulator ligand (ICOSL) and programmed cell death-1 ligand1/2 (PD-L1/2) on GC B cells regulates TCR signal activation, both positively and negatively^12^. Notably, a functional blockade or defect in negative costimulatory molecules, including programmed cell death 1 (PD1) or cytotoxic T-lymphocyte-associated protein 4 (CTLA4), induces an aberrant GC reaction and systemic autoimmunological disease^13^,^14^,^15^,^16^. These findings indicate that during T/B cell interactions, costimulatory molecules fine-tune Tfh cell differentiation, thus preventing the induction of systemic autoimmunity.

Death receptor 6 (DR6/CD358) is also known as tumour necrosis factor (TNF) receptor superfamily member 21 (TNFRSF21)^17^. The TNFRSF includes costimulatory molecules such as CD40, CD30, Herpes virus entry mediator (HVEM), 4-1BB, OX40, CD27, DR3, and glucocorticoid-induced TNFR-related protein (GITR)^18^. In a previous report, mice with a targeted deletion of the *Tnfrsf21* gene (encoding DR6) exhibited hyper production of immunoglobulins after antigen stimulation^19^, and DR6 deficiency in peripheral T cells enhances the production of cytokines for facilitating B cell activation and differentiation, as well as the antigen-dependent activation of transcriptional factors such as the nuclear factor of activated T cells (NFAT) or nuclear factor-kappa B (NFκB)^20^. DR6 is associated with the regulations on T cell function in several immunological diseases, including experimental autoimmune encephalomyelitis (EAE), asthma, and acute graft versus host disease in animal models^21^,^22^,^23^. DR6 is weakly expressed on resting peripheral CD4^+^ T cells and upregulated in response to TCR stimulation^24^. Importantly, the association of *TNFRSF21* gene induction with disease progression was reported in SLE patients^25^,^26^. Although the molecular mechanism of action, including its immunological ligand, is unknown, DR6 may have a critical role in autoimmune disease progression.

Syndecan-1 is a glycosylated type-I transmembrane protein. In *in vitro* experiments, syndecan-1 binds to various soluble proteins via its attached oligosaccharide chains. Therefore, syndecan-1 may act as a scaffold for soluble factors, inducing the accumulation of inflammatory cells in localized inflammation^27^. By contrast, several studies suggest that syndecan-1 has a suppressive function on the progression of immunological diseases. Similar to DR6 deficiency, syndecan-1 deficiency increases the severity of disease, lymphocyte activation, as well as production of antigen-specific plasma cells and immunoglobulins in the peripheral lymphoid organs of EAE, immune complex-mediated murine nephritis, and allergic lung inflammation animal models^28^,^29^,^30^. These findings suggest the presence of an undefined regulatory function of syndecan-1 in peripheral lymphocyte activation^27^. The amount of syndecan-1shed into the peripheral blood has been shown to correlate with disease severity in SLE patients^31^,^32^. Although most CD19^+^ B cells do not express syndecan-1, CD19^+^ autoreactive pre-plasma cells express syndecan-1 in lupus-prone mice and SLE patients^32^,^33^. These findings suggest that syndecan-1 is also engaged in the pathogenesis of autoimmune diseases. However, the details of the suppressive function of syndecan-1, particularly on pathogenic lymphocyte maturation and activation, are unclear^27^.

Here we show an increase in DR6 expression on Tfh cells in lupus-prone mice. Importantly, we identify syndecan-1 as a binding partner for DR6, and we clarify whether and how this dysregulated DR6/syndecan-1 interaction leads to autoimmunity in lupus-prone mice.

## Results

### Induction of DR6^+^ CD4^+^ T cells in lupus-prone mice

Recent independent studies have reported that upregulation of the *TNFRSF21* gene is associated with disease prognosis by genome-wide gene expression analysis using peripheral mononuclear cells obtained from SLE patients^25^,^26^. To explore the details of the association between DR6 and disease, we generated a rat anti-DR6 monoclonal antibody (clone 25-1, IgG1). The specificity of 25-1 antibody (25-1 Ab) is demonstrated in Supplementary Fig. 1. Briefly, 25-1 Ab bound DR6 stably expressed on L929 cells (L929-mDR6) (Supplementary Fig. 1a). 25-1 Abs specifically bound to DR6, but not to other TNFRSF molecules that were expressed on the cell surface (Supplementary Fig. 1b). 25-1 Abs could also bind to endogenous DR6, expressed by murine T cell hybridoma (DO11.10-T) cells (Supplementary Fig. 1c, left panel), and this reactivity was lost after *Tnfrsf21* gene-specific knockout based on the CRISPR-Cas9 system (G510-8-7 or -10-4) (Supplementary Fig. 1c, middle and right panels), indicating that 25-1 Abs react with endogenous DR6 but not the other proteins expressed on the cell (Supplementary Fig. 1c,d). We used 25-1 Abs to detect mouse DR6 expression hereafter.

**Figure 1:**
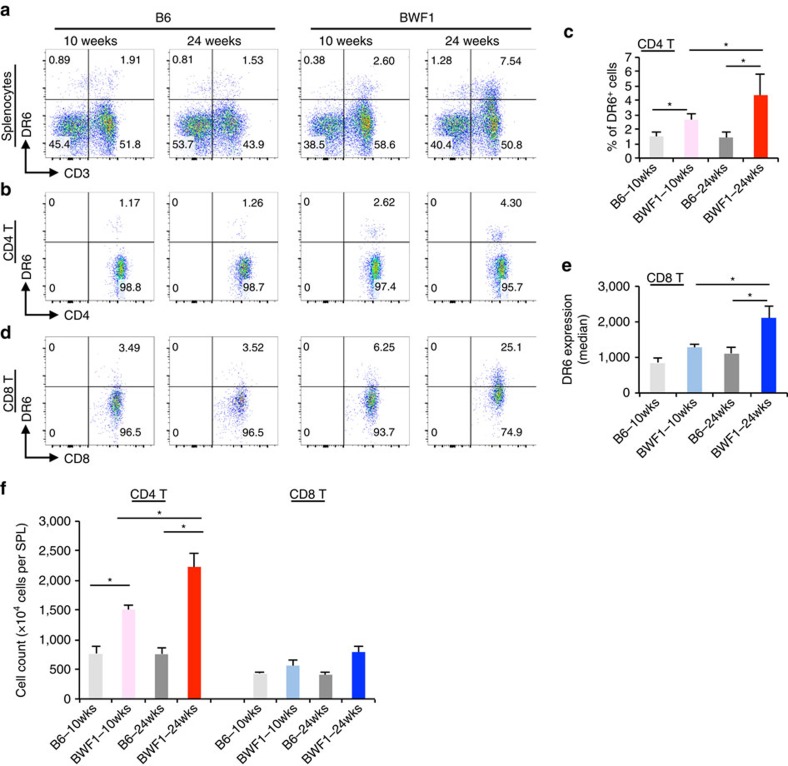
Disease-specific induction of DR6^+^ CD4^+^ T cells in lupus-prone BWF1 mice. Total splenocytes obtained from the indicated mice were stained with anti-DR6 specific monoclonal antibody (clone 25-1) with anti-CD3, CD8 and CD4 specific Abs. (**a**) Total splenocytes (gated as live singlet cells shown in Supplementary Fig. 15a) were analysed on CD3 versus DR6 dot plot. Numbers in the dot plots indicates percentage of the cells gated. (**b**) CD4^+^ T cells (CD3^+^ CD8^−^ CD4^+^) of the total splenocytes were also analysed on CD4 versus DR6 dot plots. (**c**) Quantification of the percentage of DR6^+^ cells in the CD4^+^ T cells. Error bars represent standard deviation (s.d.) (*n*=5 per group). (**d**) CD8^+^ T cells (CD3^+^ CD8^+^ CD4^−^) of splenocytes of the indicated mice were analysed on CD8 versus DR6 dot plots. (**e**) DR6 expression (median of fluorescent intensity, MFI) on CD8^+^ T cells was also quantified. (**f**) Actual numbers of total CD4^+^ T or CD8^+^ T cells (detected as shown in Supplementary Fig. 15b) were also counted in splenocytes obtained from these mice. Error bar: s.d. (*n*=5 per group). Asterisks indicate statistical significance (*P*<0.05, Student's *t*-test).

Splenocytes were obtained from anti-DNA Ab negative (10 weeks of age) and positive (24 weeks of age) female New Zealand Black × New Zealand White F1 (BWF1) mice and age- and sex matched healthy B6 mice. Interestingly, we identified the induction of DR6-expressing cells in splenocytes obtained from BWF1 but not B6 mice. The majority of DR6^+^ cells were CD3^+^ T cells in all mice that were tested. DR6^+^ cells were increased in an age-dependent fashion in BWF1 mice (Fig. 1a). Interestingly, a unique CD4^+^ subset expressing DR6 was observed in autoreactive Ab positive lupus-prone BWF1 (aged 24 weeks) but not in autoreactive Ab negative B6 mice (aged 10 and 24 weeks; Fig. 1b,c). Most of the CD8^+^ T cells also expressed DR6 in BWF1 mice (Fig. 1d,e). While CD4^+^ T cells of BWF1 mice markedly expanded with disease progression, CD8^+^ T cells were minimally expanded in the mice (Fig. 1f). Therefore, we decided to further define which subpopulation of CD4^+^ T cells expressed DR6.

### DR6 expression on Tfh cells of lupus-prone mice

**Figure 2:**
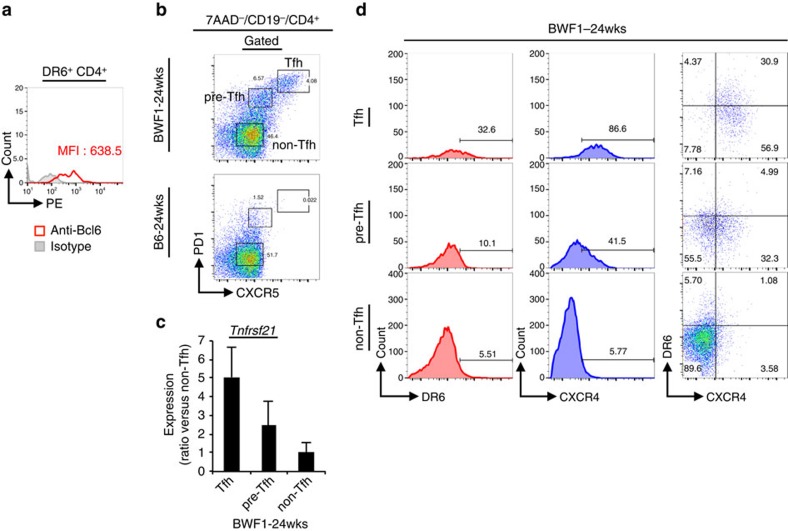
DR6 expression on Tfh cells of lupus-prone BWF1 mice. (**a**) DR6^+^ CD4^+^ cells of 24-week-old female BWF1 mice were stained with PE-conjugated anti-Bcl6 or isotype matched control Ab. The antibody specific signals of the cells are shown in histogram. (**b**) 7AAD^−^ CD19^−^ CD4^+^ splenocytes obtained from the indicated mice were analysed on CXCR5 versus PD1 dot plots for recognizing Tfh (PD1^high^ CXCR5^high^) and pre-Tfh (PD1^int^ CXCR5^int^) or non-Tfh (PD1^−^ CXCR5^−^ ) cells. (**c**) The Tfh cell populations of 24-week-old female BWF1 mice were sorted and then *Tnfrsf21* mRNA expression in these fractionated cells was analysed by quantitative PCR (qPCR). Error bar: s.d. (*n*=5 per group). (**d**) Both DR6 and CXCR4 expressions of the indicated Tfh cell populations that were detected as in **b** are also shown. For detecting Tfh cell populations, gate strategy that was shown in Supplementary Fig. 15c was used in **b**–**d**.

Follicular helper T (Tfh) cells expand and promote disease progression in lupus-prone mice. Therefore, we asked whether DR6 expression was associated with Tfh cells in lupus-prone BWF1 mice. Thus, we characterized Bcl6 expression of DR6^+^ CD4^+^ cells by intracellular staining since Bcl6 is a key transcriptional factor for Tfh cells (Fig. 2a). Interestingly, we detected anti-Bcl6 antibody specific signal in DR6^+^ CD4^+^ splenocytes isolated from 24-week-old BWF1 mice (Fig. 2a). We also analysed DR6 expression among Tfh cells that expanded in lupus-prone BWF1 mice. The expanded Tfh cells were detected by their expression of CXCR5 and PD1 among 7AAD^−^ (live) CD19^−^ CD4^+^ splenocytes of 24-week-old female BWF1 mice (Fig. 2b, upper panel), but not in age- and sex matched B6 mice (Fig. 2b, lower panel). By their CXCR5 and PD1 expression levels, the expanded Tfh cells could also be separated into three populations; CXCR5^high^/PD1^high^ (mature Tfh cells) and CXCR5^int^/PD1^int^ (pre-Tfh cells) or CXCR5^−^/PD1^−^ (non-Tfh cells). To determine *Tnfrsf21* gene expression of these three Tfh cell populations, we sorted these cells from splenocytes of 24-week-old BWF1 mice. We then detected expression of the *Tnfrsf21* gene in these isolated cells by quantitative PCR (Fig. 2c). *Tnfrsf21* mRNA expression was detected in each cell population isolated from BWF1 mice. The expression was highest in Tfh cells; intermediate in pre-Tfh and low in non-Tfh cells. We also analysed the DR6 protein expression of these Tfh cell populations. In flow cytometrical assays, DR6 expressing cells were observed in each Tfh cell population. The frequency of DR6^+^ cells was highest in Tfh cells, intermediate in pre-Tfh cells and rare in non-Tfh cells (Fig. 2d, left panels). The DR6 expression on Tfh cells correlated with the expression of another Tfh-associated surface molecule, CXCR4, by the cells (Fig. 2d, middle and right panels). These findings indicated that DR6 is expressed on Tfh cells that expand in lupus-prone BWF1 mice.

### Identification of syndecan-1 as a ligand for DR6

**Figure 3:**
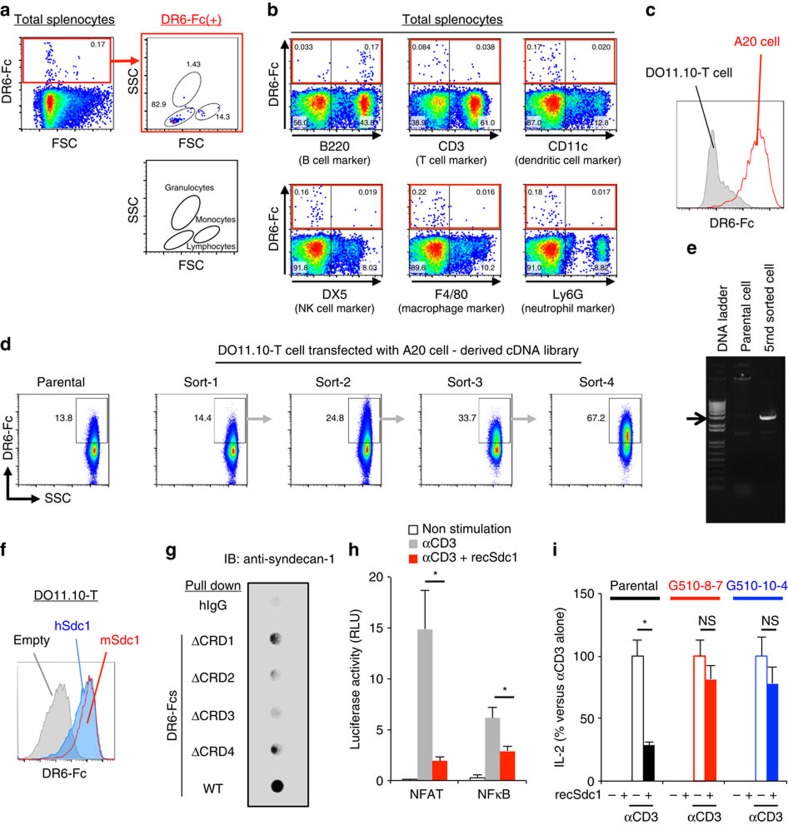
Identification of syndecan-1 as a functional binding partner for the DR6 ectodomain. (**a**; left panel) Total splenocytes of B6 mice were stained with biotinylated murine DR6-Fc plus allophycocyanin-conjugated streptavidin. (upper right panel) Cells bound to DR6-Fc among live singlet splenocytes were analysed on FSC versus SSC dot plots. (lower right panel) The gating shown in the FSC versus SSC dot plot is also schematically explained. (**b**) Total splenocytes were also stained with DR6-Fc and antibody for detecting the indicated cell type. (**c**) A20 or DO11.10-T cells were stained with DR6-Fc. DR6-Fc specific fluorescent intensity of these cell lines is shown. (**d**) DO11.10-T cells transfected with a cDNA library derived from A20 cells were stained with DR6-Fc. The transfectants bound to DR6-Fc were collected by four rounds of sorting and expansion. (**e**) After the fifth round of sorting, the cDNA library inserted in genomic DNA of the selected transfectants was isolated as a single cDNA fragment (∼3,000 bp). (**f**) Intensity of DR6-Fc staining on DO11.10-T cells ectopically expressing *Syndecan-1* of mouse (mSdc1) or human (hSdc1) or empty plasmid. (**g**) Endogenous syndecan-1 of A20 cells was precipitated with the indicated Fc-fusion proteins and detected as described in Methods section. WT means Fc protein fused with the extracellular region of DR6 wild type, ΔCRD1 means the fusion protein lacking CRD1, ΔCRD2, lacking CRD2, ΔCRD3, lacking CRD3 or ΔCRD4, lacking CRD4. (**h**) DO11.10-T cells were transfected with the indicated reporter plasmid with reference plasmid. The cells were then stimulated with the indicated combination of recombinant syndecan-1 protein (recSdc1, 30 nM) and anti-CD3 Ab (10 μg ml^−1^). Six hours after the stimulation, reporter activities of the cells were assessed. Error bar: s.d. (*n*=3 per group). (**i**) Parental DO11.10-T or its DR6-knockout clone (G510-8-7 or G510-10-4) was stimulated as described in **h**. Twelve hours after the stimulation, IL-2 production of the cells was assessed as described in Methods section. Error bar: s.d. (*n*=3 per group). Details of the DR6 knock out single cell clones is also shown in Supplementary Fig. 1c,d. Asterisks or NS in each panel indicate statistical significance (*P*<0.05, Student's *t*-test) or not significance, respectively.

To understand in detail the function of DR6 in immunological events, specific ligand(s) for DR6 required discovery. To identify the DR6 ligand(s), we generated a soluble fusion protein consisting of an extracellular domain of murine DR6 and the Fc portion of human IgG (DR6-Fc). We observed a small portion of the cells bound to DR6-Fc in total splenocytes obtained from B6 mice (Fig. 3a, red box in left panel). Most of the cells bound to DR6-Fc were detected in the lymphocytes gate on a SSC versus FSC dot plot (Fig. 3a, right panels). We also stained total splenocytes with DR6-Fc and a specific antibody against the indicated cell type marker. The cells bound to DR6-Fc were detected with an antibody against a surface marker for B cells, B220, but not for other cell populations tested (Fig. 3b, red coloured box). The murine A20 B cell line, but not DO11.10-T cell hybridoma, preferentially bound to DR6-Fc (Fig. 3c). Thus, we constructed expression plasmids of full-length complementary DNA (cDNA) library obtained from DR6-Fc-positive A20 cells and retrovirally transfected the plasmids into DR6-Fc-negative DO11.10-T cells. Then, we isolated the transfectants bound to DR6-Fc during four rounds of sorting and culturing expansions (Fig. 3d). After the fifth round, we recovered a single library-specific cDNA fragment (approximately 3,000 bp in length) inserted in genomic DNA of the isolated transfectants (Fig. 3e). Surprisingly, the fragment encoded the sequence for the full-length mRNA of murine *Syndecan-1* (*Sdc1*). The binding of syndecan-1 to DR6 was confirmed by human and mouse syndecan-1 ectopically expressed on DO11.10T cells binding to the DR6 ectodomain (Fig. 3f).

We then analysed the molecular specificity of the interaction between syndecan-1 and DR6 (Supplementary Fig. 2). We retrovirally transfected plasmids expressing non-specific (shLuc) or *Syndecan-1*-specific (shmSdc1) shRNAs into A20 cells. Using flow cytometry, we confirmed the effective knockdown of syndecan-1 protein expression mediated by shmSdc1, but not shLuc, in A20 cells (Supplementary Fig. 2a). We also confirmed that shmSdc1 treatment did not affect B220 expression of the cells (Supplementary Fig. 2b). In a further flow cytometrical assay using DR6-Fc, shmSdc1 but not shLuc treatment dramatically reduced the binding of DR6-Fc to A20 cells (Supplementary Fig. 2c). In the same assay using the indicated recombinant Fc proteins, syndecan-1 expressed on A20 cells preferentially bound to DR6-Fc compared with Fc proteins obtained from the other TNFRSF molecules (Supplementary Fig. 2d). This syndecan-1 specific interaction of DR6 was consistent with the observation that the genome-wide screening identified only syndecan-1 as a binding molecule for the DR6-ectodomain (Fig. 3e).

We also analysed binding specificity within the molecular structures of DR6 (Fig. 3g). Four cysteine-rich domains (CRDs) are conserved in the extracellular region of DR6 and are involved in ligand binding^17^. We generated recombinant Fc proteins fused with DR6-extracellular domain lacking CRD1 (ΔCRD1), CRD2 (ΔCRD2), CRD3 (ΔCRD3) or CRD4 (ΔCRD4). Then, these recombinant proteins were pulled down with syndecan-1 endogenously expressed on A20 cells. The precipitated syndecan-1 was detected with an anti-syndecan-1 Ab. Among the tested Fc proteins, the mutants lacking CRD2 or CRD3 were not capable of precipitating syndecan-1. The observed dependence of the interaction on CRD2 and -3 is in agreement with the previous observations that both CRD2 and -3 of DR6 are positively charged for its ligand binding^34^.

We further demonstrated a molecular function of syndecan-1/DR6 interaction in T cell activation (Fig. 3h). DO11.10-T cells were electroporated with the indicated transcription factor-dependent reporter plasmids. Then, the cells were stimulated with or without recombinant syndecan-1 (recSdc1) under activation of TCR signalling mediated by anti-CD3 Ab. CD3 cross-linking dramatically activated NFAT in the cells (Fig. 3h, gray in left columns). Interestingly, the recSdc1 stimulation suppressed NFAT activation (Fig. 3h, red versus gray in left columns). The suppressive effect of recSdc1 was also observed in the reporter assay detecting NFκB activation (Fig. 3h, right columns). We also demonstrated the suppressive function of recSdc1 by assessing IL-2 production, which depends on NFAT and NFκB activation (Fig. 3i). Parental DO11.10T cells or their DR6 deficient clones (Supplementary Fig. 1c,d) were stimulated with the indicated combinations of anti-CD3 Ab and recSdc1. Parental DO11.10T cells produced IL-2 in response to anti-CD3 Ab, whereas the cells produced decreased IL-2 in the presence of recSdc1 (Fig. 3i, opened compared with closed column in black coloured columns). Interestingly, DR6 deficient DO11.10T cell clones produced IL-2 in the presence or absence of recSdc1, indicating that the suppressive effect of recSdc1 depends on DR6 expression by the cells (Fig. 3i, opened compared with closed in red or blue columns). These findings demonstrate that syndecan-1 is a specific binding partner for DR6 and the interaction negatively regulates NFAT and NFκB activation during TCR activation.

### Syndecan-1 on autoreactive germinal centre B cells

**Figure 4:**
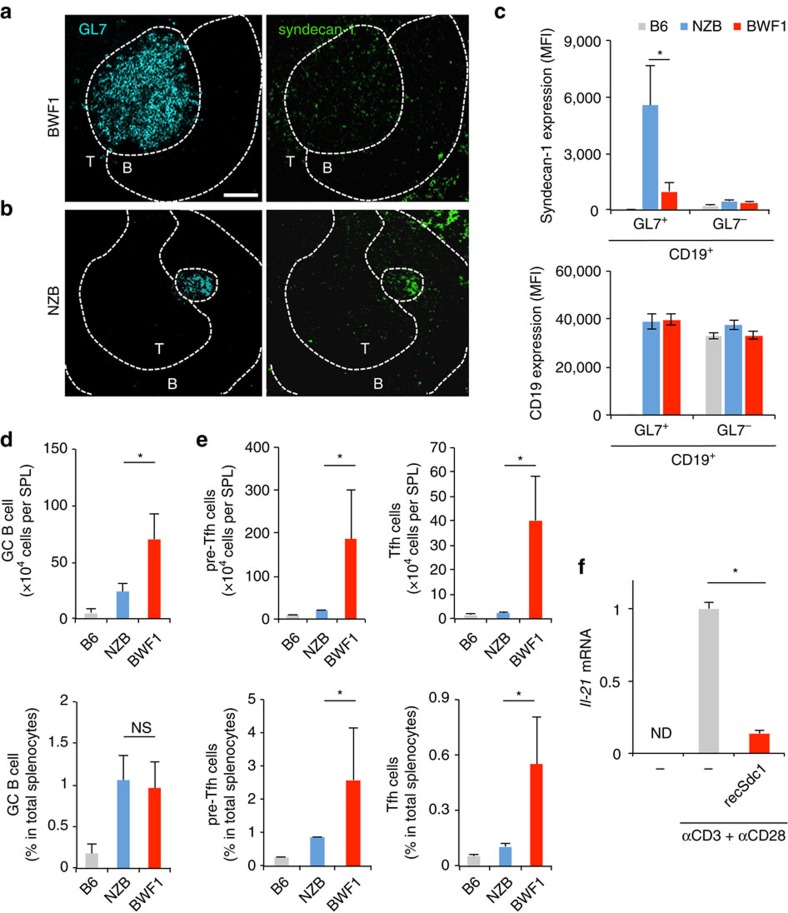
Syndecan-1 induction on GC B cells in lupus-prone mice. (**a**–**e**) Spleens were isolated from 24-week-old female BWF1, NZB or B6 mice. Splenic cryosections obtained from the BWF1 (**a**) or NZB (**b**) mice were stained by anti-GL7 (left panel) and syndecan-1 (right panel) specific antibodies. Additional anti-CD4 Ab staining was also used for identifying the border between B and T cell zone. Representative images of at least 3 independent fields per slide are shown. B, B cell zone; T, T cell zone. Dashed line indicates the border between each region. Bar=100 μm. (**c**) GL7^+^ CD19^+^ and GL7^−^ CD19^+^ B cell populations obtained from the indicated mice were detected by flow cytometry with the gate strategy that is shown in Supplementary Fig. 15d. The expression levels of syndecan-1 (upper panel) and CD19 (lower panel) on the detected B cell populations were shown. Error bar: s.d. (*n*=3 for B6, 5 for NZB, 5 for BWF1 per group). (**d**) Mean number (upper panel) or percentage (lower) of the GL7^+^ CD19^+^ GC B cell in live total splenocytes obtained from the indicated mice. Error bar: s.d. (*n*=3 for B6, 5 for NZB, 5 for BWF1 per group). (**e**) The number (upper panel) or percent (lower) of pre-Tfh (left) or Tfh cells (right) of live total splenocytes from the indicated mice are also analysed with the gate strategy shown in Supplementary Fig. 15c. Error bar: s.d. (*n*=5 per group). (**f**) CD3^+^ CD4^+^ PD1^high/int^ CXCR5^high/int^ CXCR4^+^ cells were isolated from 24-week-old female BWF1 mice. The cells were stimulated with the indicated combinations of recSdc1 (30 nM), anti-CD3 Ab (10 μg ml^−1^) and anti-CD28 Ab (10 μg ml^−1^). Seventy-two hours after the indicated stimulations, *Il-21* mRNA production in the cells was analysed by qPCR. Error bar: s.d. (*n*=3 per group). ND, not detected. Asterisks in each panel indicate statistical significance (*P*<0.05, Student's *t*-test).

We next considered whether syndecan-1 was associated with a specific B cell population, germinal centre (GC) B cells, because the GC B cell provides critical signals for complete maturation and expansion of Tfh cells in lupus-prone mice^9^,^10^,^35^,^36^. We isolated spleens from non-immunized 24-week-old female BWF1 and NZB mice. On the splenic cryosections of BWF1 mice, anti-GL7 Ab stained the GC cells, which were markedly expanded on the border between the T- and B-cell zones (Fig. 4a, cyan in left panel). Interestingly, the GL7^+^ GC cells were also stained with anti-syndecan-1 Ab (Fig. 4a, green in right panel). In another lupus-prone mouse strain, NZB mice, the GC cells (Fig. 4b, cyan) also expressed syndecan-1 (Fig. 4b, green). The same results were obtained from other sections (Supplementary Fig. 3). Comparing the observations obtained from BWF1 (Fig. 4a) with NZB mice (Fig. 4b), we found some differences; (1) the level of syndecan-1 expression on GC cells was higher in NZB than BWF1 mice, and (2) the size of expanded GC cells was smaller in NZB than BWF1 mice. To confirm the former, we assessed the expression level of syndecan-1 on GL7^+^ CD19^+^ GC B cells using flow cytometry. As expected, the level of syndecan-1 expression on GL7^+^ CD19^+^ GC B cells of NZB mice was higher than that of BWF1 mice (Fig. 4c, upper panel). The levels of syndecan-1 expression of GL7^−^ CD19^+^ non-GC B cells were low and comparable between lupus-prone and healthy B6 mice (Fig. 4c, upper panel), although CD19 expression level was comparable among these cells (Fig. 4c, lower panel). To confirm the latter, we calculated the numbers and percentages of GC B cells in these mice strains. While GL7^+^ CD19^+^ GC B cells hardly developed in healthy non-immunized B6 mice, the cells expanded in lupus-prone NZB and BWF1 mice (Fig. 4d, upper panel). The number of the GC B cells was significantly lower in the spleen of NZB mice compared with BWF1 mice (Fig. 4d, upper panel). The percentage of GC B cells was also comparable among these lupus-prone mice strains (Fig. 4d, lower panel). Therefore, lupus-specific expansion of GC B cell should be restricted in NZB, although the GC B cells developed in both NZB and BWF1 mice. Further analysis also showed the NZB specific restriction of the expansion of both pre-Tfh (Fig. 4e, left panels) and Tfh cells (Fig. 4e, right panels). Therefore, we wondered whether syndecan-1 was associated with the regulation of Tfh cells in lupus-prone mice. To answer this question, we isolated DR6-expressing Tfh cells from BWF1 mice and stimulated the cells with recSdc1 in conjunction with TCR activation *in vitro*. Surprisingly, recSdc1 stimulation (at 30 nM) suppressed *Il-21* production by the Tfh cells during activation (Fig. 4f). The recSdc1-mediated suppressive effect was not due to a cytotoxic effect on the Tfh cells because 30 nM recSdc1 did not stimulate cell death induction in DO11.10T cells (Supplementary Fig. 4) or the Tfh cells (Supplementary Fig. 5). These findings indicate that syndecan-1 was expressed on GC B cells of lupus-prone mice.

### Suppression of DR6^+^ Tfh cells delays disease progression

**Figure 5:**
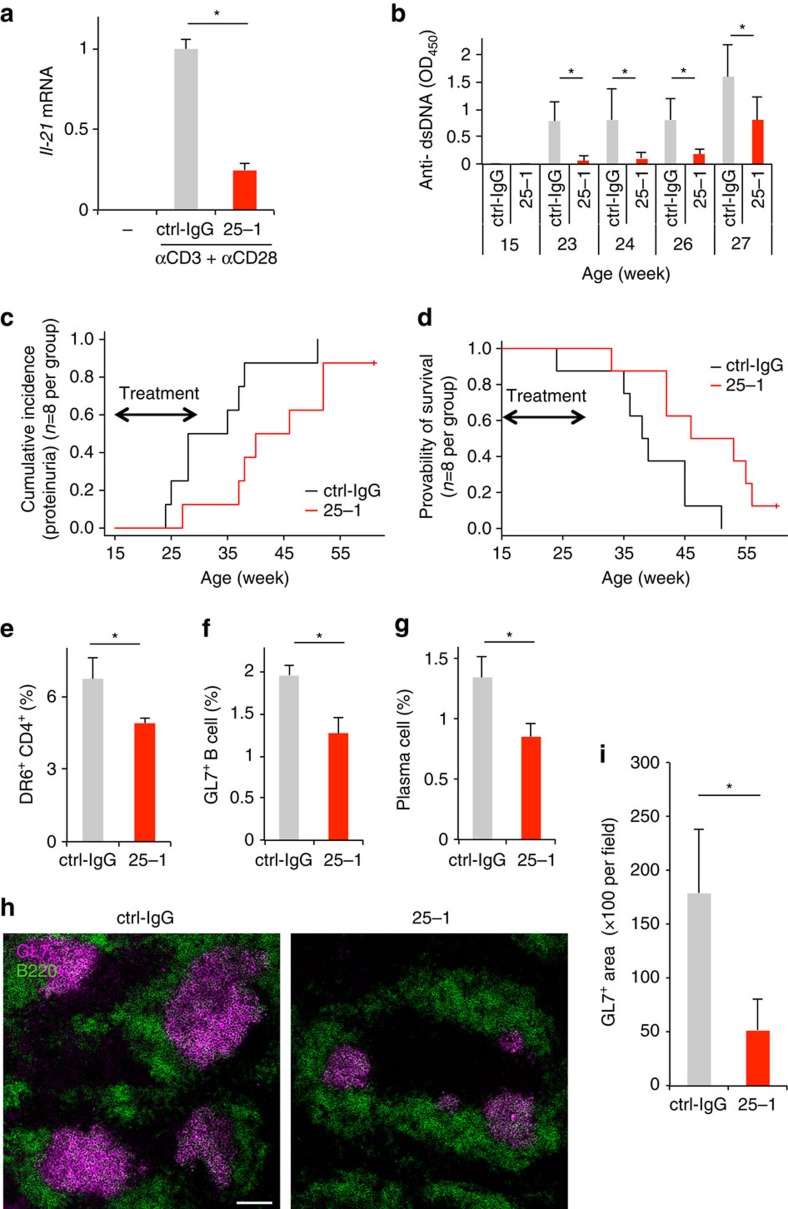
Anti-DR6-specific antibody delays lupus-like syndrome progress in lupus-prone mice. (**a**) Effect of 25-1 Ab (25-1) or nonspecific rat IgG (ctrl-IgG; 10 μg ml^−1^) administration on *Il-21* production of CD3^+^ CD4^+^ PD1^high/int^ CXCR5^high/int^ CXCR4^+^ cells was assessed as described in Fig. 4f. (**b**–**d**) 15-week-old female BWF1 mice were intraperitoneally administered nonspecific control rat IgG (ctrl-IgG) or 25-1 Ab (300 μg per mouse every 2 days, from 15 to 27 weeks old; *n*=8 per group). (**b**) anti-dsDNA Ab production in sera of these mice was assessed by ELISA. Error bar: s.d. (*n*=8 per group). (**c**) Percentages of proteinuria positive (>100 mg protein per dl) mice in each group are shown (*P*=0.0314 by Gray test). (**d**) The survival rate of these groups is also shown (*P*=0.0251 by Log-rank test). (**e**–**i**) Twenty-four-week-old proteinuria-positive female BWF1 mice were treated as described above (until 32 weeks old) (*n*=4 per group). Two days after the last administration, frequency of DR6^+^ cells in CD4^+^ T cells (**e**), GL7^+^ GC B cell in B220^+^ B cells (**f**) or syndecan-1^high^ B220^−^ plasma cells in live total splenocytes (**g**) obtained from these mice was assessed by flow cytometry. Gate strategies that were used for identifying each cell population were shown in Supplementary Fig. 15a–e. Error bar: s.d. (*n*=4 per group). (**h**) Splenic cryosections obtained from these mice were stained by GL7 Ab for detecting GCs (magenta). The sections were also stained by anti-B220 Ab (green) for detecting B cell area. Bar, 100 μm. The results obtained from the other sections are also represented in Supplementary Fig. 9. (**i**) Area of GL7^+^ in each section was also calculated. Error bar, s.d. (*n*=4–6 per section). Asterisks in each panel indicate statistical significance (*P*<0.05, Student's *t*-test).

We found that 25-1 Ab is capable of DR6 cross-linking in *in vitro* experiments (Supplementary Fig. 6, the details are described in the legend). In addition, 25-1 Ab suppressed *Il-21* production by DR6-expressing Tfh cells isolated from BWF1 mice (Fig. 5a). This effect was not due to cytotoxicity because 25-1 Ab did not affect cell death induction in DO11.10-T (Supplementary Fig. 7) or isolated DR6-expressing Tfh cells (Supplementary Fig. 8). These observations prompted us to test the effect of 25-1 Ab on disease progression in BWF1 mice. We administered 25-1 Ab (25-1) or nonspecific rat IgG as a control (ctrl-IgG) to 15-week-old BWF1 mice. Surprisingly, the 25-1 Ab administrations significantly reduced serum anti-dsDNA Ab production (Fig. 5b), the percentage of mice with proteinuria (as a marker for kidney dysfunction; Fig. 5c), and improved survival (Fig. 5d) compared with the ctrl-IgG administration. In addition, 25-1 Ab administration significantly reduced the induction of DR6^+^ CD4^+^ cells (Fig. 5e), GC B cells (Fig. 5f) and plasma cells in BWF1 mice (Fig. 5g). The splenic cryosections also showed 25-1 Ab-mediated suppressive effects on GC formation (Fig. 5h,i, the results obtained from the other sections represented in Supplementary Fig. 9). In addition, we did not observe a significant effect of 25-1 Ab on antibody dependent cellular cytotoxicity (ADCC, Supplementary Fig. 10a) or complement dependent cytotoxicity (CDC, Supplementary Fig. 10b). These findings indicate that regulation of DR6 delays disease progression in lupus-prone mice.

## Discussion

In this current study, we found a disease-specific induction of DR6^+^ T cells in lupus-prone BWF1 mice. In particular, the induction of DR6^+^ CD4^+^ T cells correlated with age- and genetic background-dependent disease progression and prognosis in lupus-prone mice. A large proportion of CD8^+^ T cells also expressed DR6. However, the expansion of these CD8^+^ T cells was suppressed or not promoted in lupus-prone mice. Therefore, we further analysed DR6 expressing CD4^+^ T cells. DR6^+^ CD4^+^ cells expressed *Il-21* and *Bcl6*. The expression levels were lower than those observed in DR6^low^ Tfh cells (Supplementary Fig. 11). The up-regulated expression of DR6 on the T cell surface-regulated the threshold of DR6 activation to suppress activation of TCR signalling in the cells. However, the up-regulated DR6 itself is insufficient for complete prevention of Tfh cells in lupus-prone BWF1 mice, because of the low amount of DR6 ligand on the surface of GC B cells that are the direct counter partners of the Tfh cells. In addition, recent studies have reported that CD4^+^ T cell populations exhibit disease-associated characteristics caused by several SLE-associated genetic mutations on murine chromosomes 1, 5, 6, 7 and 18, leading to high activation and expansion of CD4^+^ T cell populations in lupus-prone mice^37^,^38^,^39^,^40^. Although the murine *Tnfrsf21* gene (on chromosome 17) is not in murine SLE-associated loci, the unique characteristics of the lupus CD4^+^ T cell populations emphasize the importance of DR6 functions in the regulation of CD4^+^ T cells in lupus-prone mice.

Upregulation of DR6 expression was observed in Tfh cell populations of lupus-prone BWF1 mice. In contrast to lupus-prone mice, DR6 expression was low in Tfh cells of DNP-KLH- immunized mice (Supplementary Fig. 12a,b, vertical axes). CXCR4 (Supplementary Fig. 12b, horizontal axes) or Bcl6 (Supplementary Fig. 12c) expression in these cells was also low compared with that in Tfh cells of BWF1 mice. Collectively, Tfh cells of lupus-prone mice manifested their pathological characteristics compared with Tfh cells of healthy mice as expected, considering that lupus CD4^+^ T cell populations have a high sensitivity to TCR activation^37^,^39^. The TCR signal promoted high DR6 induction on Tfh cells of lupus-prone mice (Supplementary Fig. 13a,b). Recently, these pathological characteristics of lupus CD4^+^ T cells were shown to be caused by aberrant metabolic regulations, and are crucial for spontaneous induction of Tfh cell generation, GC formation, and disease symptoms in lupus-prone mice^38^. Interestingly, such aberrant regulation of lupus CD4^+^ T cells is not associated with humoral responses against foreign antigens^38^. Therefore, the pathological cellular characteristics might involve high DR6 induction on auto reactive Tfh cells of severe lupus-prone BWF1 mice.

We characterized syndecan-1 as a binding partner for DR6. Recombinant syndecan-1 stimulation was not capable of inducing cell death, except when it was highly concentrated (Supplementary Fig. 4). This weak apoptotic effect of syndecan-1 stimulation is consistent with the weak apoptotic capability of DR6 compared with that of the other TNFRSF molecules, such as death receptor-4 (DR4)^17^ and -3 (DR3)^41^. This weak apoptotic activity of DR6 might depend on its low affinities of binding to Fas-associated protein with death domain (FADD) and TNFR-associated death domain protein (TRADD), which are key components for apoptotic caspase activation^17^. In addition, DR6 receptor cross-linking did not affect caspase activation in the cultured T-cell line (Supplementary Fig. 14). Meanwhile, syndecan-1/DR6 interaction dramatically suppressed the activation of TCR signalling, which activates NFκB and NFAT in the present study. In addition, DR6 gene deficiency in lymphocytes leads to hyper activation of NFκB or NFAT^19^,^20^. These findings and our data suggest that DR6 regulates lymphocyte activation by its negative regulation to NFκB and NFAT signalling rather than apoptotic function. However, the molecular mechanisms of this DR6-mediated suppression have yet to be elucidated.

Syndecan-1 has multiple functions in immunological events and disease progression^27^. Syndecan-1 can attach to several chemokines, growth factors, and cytokines, suggesting that it has a scaffolding function for the accumulation of inflammatory cells in localized inflammation. Several knockout studies also suggest that syndecan-1 regulates antibody production. Syndecan-1 deficiency induces pathogenic antigen (myelin oligodendrocyte glycoprotein)-specific plasma cells in an EAE model^28^. Syndecan-1 deficiency also causes high immunoglobulin production in an Ab-dependent nephritis model^29^. Importantly, DR6 is also associated with negative regulation of peripheral lymphocyte activation in syndecan-1-associated immunological disease models such as EAE^21^, lung inflammation^22^, and lupus-like disease (the present study). These findings suggest an association of DR6/syndecan-1 interaction with the progression of several immunological diseases.

The 25-1 Ab was capable of DR6 cross-linking. Recombinant syndecan-1, as well as 25-1 Ab treatment, suppressed *Il-21* production by Tfh cells. Therefore, the antibody should mimic the function of syndecan-1 that was reduced in BWF1 mice. IL-21 production by Tfh cells is critical for both maintenance and development of GC B cells^42^. Although the importance of IL-21 production in Tfh cell development is controversial^43^, GC B/Tfh cell interaction is important for full-maturation and maintenance of Tfh cells and GC formation^9^,^44^,^45^. Therefore, 25-1 Ab or syndecan-1 should fine-tune the outcome of GC B/Tfh cell interactions, to prevent autoantibody production and disease progression in BWF1 mice. Moreover, although 25-1 Ab preferentially bound to CD3^+^ T cells, other cells binding 25-1 Ab were also observed in total splenocytes obtained from BWF1 mice. Several reports have shown DR6 expression on B cells, dendritic cells and macrophages^19^,^46^,^47^. In addition, these cells are associated with GC reactions after immunization in healthy mice, as well as the disease progression of lupus-prone mice. Therefore, further studies will be needed to investigate the role of DR6/syndecan-1 interaction in the regulation of these cells in the immunization as well as the disease progression.

Taken together, in the current study, we identified a novel regulatory mechanism that depends on the interaction of DR6 with syndecan-1 for preventing expansion and activation of Tfh cells and disease progression in lupus-prone mice. Therefore, the DR6/syndecan-1 axis should be a novel therapeutic target in autoimmune disease.

## Methods

### Mice

Female C57BL/6, New Zealand Black (NZB), or NZB × New Zealand White F1 mice were purchased from SLC Inc. (Shizuoka, Japan). These mice were housed in specific pathogen-free conditions. All mice were assigned randomly to experimental groups. We performed animal care and experiments in accordance with the guidelines and approval of the Animal Care and Use Committee of Hokkaido University.

### Cells

Murine fibrosarcoma L929, human embryonic fibroblast (HEK) 293T cells, and retrovirus packaging cells (PLAT-E, kindly provided by Dr Kitamura T) were maintained in Dulbecco's modified Eagle's medium containing 10% fetal calf serum (FCS) at 37 °C in 5% CO_2_. Murine T cell hybridoma DO11.10-T and murine lymphoma A20 were also maintained in RPMI-1640 medium containing 10% FCS. All cell lines were routinely tested for mycoplasma.

### Generation of recombinant proteins

For construction of DR6-Fc, cDNA encoding the extracellular region of DR6 (mouse: from +1_+350 aa) was fused with the Fc region of human IgG, which was mutated at the amino acid position of E233P, L234A, and L235A for preventing binding with mouse Fc receptors, and inserted into pcDNA3 (Life Technologies, Carlsbad, CA). The resulted plasmid was transfected into HEK293T cells with lipofectamine 2000 (Life Technologies). The recombinant protein secreted in the cultured supernatant was purified by protein-A sepharose column^48^. Biotinylation of the proteins was performed using the EZ-Link NHS-PEO Solid Phase Biotinylation Kit (Life Technologies), according to the manufacturer's instructions. For the generation of recombinant mouse syndecan-1 protein (recSdc1), the cDNA encoding ectodomain of mouse syndecan-1 (+1_+251 aa) was inserted into pcDNA3.1 myc/his ver.B (Life Technologies). The production of recSdc1 that was fused with hexamer His tags was achieved in HEK293T cells as described above. RecSdc1 in culture medium was purified using Ni-sepharose column (HisTrap HP, GE Healthcare, Piscataway, NJ).

### Generation of anti-DR6 specific monoclonal antibody

Hybridomas were generated from splenocytes of Lewis rats immunized with DR6-Fc and L929-mDR6 cells. Specificity of the antibody produced in the cells was assessed by enzyme-linked immunosorbent assay (ELISA) using DR6-Fc or human IgG as antigen.

### Flow cytometry

Details of antibodies used in the present study are listed in Supplementary Table 1. For analysing cell surface molecules, single-cell suspensions were stained with the indicated antibodies (the concentration of antibody is also listed in Supplementary Table 1), after Fc-block with anti-CD16/32 (clone 2.4G2, 20 μg ml^−l^, 4 °C, 15 min). After washed twice with ice-cold PBS containing 0.5% bovine serum albumin and 0.1% NaN_3_, the cells were further stained with 7AAD. Fluorescent intensities of the cells were detected on a LSR Fortessa or Canto (BD Biosciences, San Jose, CA). For identifying lymphocyte cell populations that were indicated in each figure, gate strategies were shown in Supplementary Fig. 15. For intracellular Bcl6 detection^49^, Fixable Viability Dye eFluor 780 (eBioscience, San Diego, CA) was used instead of 7AAD.

### Expression screening

Expression screening using the retrovirus-based full-length cDNA library was performed as described in our previous report^50^. Briefly, the full-length cDNA library was constructed using the In-Fusion SMARTer Directional cDNA Library construction kit (Takara Bio, Shiga, Japan) and inserted into retrovirus vector, pMXs^51^. The titer of cDNA library was >1 × 10^6^ plaque forming units per ml, and >90% of individual clones contained different cDNA fragments. pMXs containing cDNA library was retrovirally packaged in PLAT-E cells. DO11.10-T cell hybridoma (2 × 10^6^ cells) was infected with the virus particles at a multiplicity of infection=0.1. Two days after the infection, the cells were stained with biotinylated DR6-Fc plus streptavidin-allophycocyanin (APC). After two-washes, APC-positive cells were sorted by MoFlo Astrios (Beckman Coulter, Brea, CA). The sorted cells were then expanded. After five-rounds of sorting and culturing, the provirus in the sorted cells was isolated by PCR using pMXs-specific primer set.

### Stable gene expression

cDNA fragment encoding the indicated gene was cloned into pMXsIG^51^ at multiple cloning sites. The construct was retrovirally transfected into cells. Among the cells, enhanced green fluorescent protein positive cells were sorted by Moflo Astrios. The purity of the cells was confirmed as >90% by flow cytometry.

### *Tnfrsf21* knockout single cell clone

The 23 nucleotide guide sequence (5′-CCACGATGGTCGCCGGCTCTCTT-3′ ) targeting on negative strand genomic DNA locating on exon-1 of murine *Tnfrsf21* gene was designed with CRISPR direct software uploaded in http://crispr.dbcls.jp. The DNA fragment was cloned into pSpCas9(BB)-2A-Puro (PX459) ver.2 (ref. 52). The resulted plasmid was electroporated into DO11.10-T cell using Gene Pulser X-cell (Bio-Rad, Hemel Hempstead, UK) at 280 V and 950 μF. Two days after puromycin selection at 3 μg ml^−1^, single cell clones of the cells were isolated by limiting dilution. The single cell clones were selected after their re-exhibition of puromycin sensitivity (for validating plasmid exclusion) and sequencing their genomic DNA (for gene editing).

### Quantitative PCR

Quantitative PCR assay was performed using SYBR Premix Ex Taq II (Takara Bio.)^41^. Briefly, cellular total RNAs were isolated by Trizol method according to the manufacturer's instruction (Life Technologies). Reverse-transcription was carried out by using ReverTra Ace (Toyobo CO. Ltd., Osaka, Japan). Gene-specific primer sets used in the current study are shown in Supplementary Table 2. Mean of *Actb* in each sample was used as internal control.

### Site-directed mutagenesis

For generating region specific deletion mutants of DR6 (ΔCRD1; deleted the region from +42 to +105 aa, ΔCRD2; deleted the region from +106 to +145 aa, ΔCRD3; deleted the region from +146 to +185 aa or ΔCRD4; deleted the region from +186 to +212 aa), site-directed deletions were introduced into the plasmid encoding DR6-Fc using PrimeSTAR Mutagenesis kit (Takara Bio.) according to the manufacturer's instruction.

### Pull down assay

A20 cells (2.5 × 10^7^ cells) were incubated with the indicated Fc fused protein (10 μg ml^−1^) at 4 °C. Two hours after the incubation, cells were washed three times with ice-cold culture medium and lysed with the lysis buffer containing 0.2% NP-40, 10% Glycerol, 150 mM NaCl and 20 mM Tris-HCl (pH 7.4) at 4 °C. After centrifugation (20,000 × *g*, 30 min, 4 °C), the supernatant was precipitated with protein A sepharose beads (GE Healthcare) at 4 °C for 2 h. The beads were washed three times with the lysis buffer and the eluted materials were analysed by dot blot assay using anti-syndecan-1 Ab (clone 281-1, 1/1,000 diluted, Biolegend, San Diego, CA).

### Reporter gene assay

Cells (3 × 10^6^ cells per 450 μl of Opti-MEM (Life Technologies) in a 0.4-cm cuvette) were electroporated with 20 μg firefly-luciferase reporter plasmid (pGL4.26, Promega) containing binding motif for the indicated transcriptional factor plus 5 μg reference renilla-luciferase reporter plasmid (phRG-TK, Promega) as described above. Twenty-four hours after the electroporation, cells were stimulated with plate-bound anti-CD3 Ab (10 μg ml^−1^) in the presence or absence of recSdc1 (30 nM). Six hours after the stimulation, the luciferase activities of the cells were assessed by dual-Glo luciferase assay (Promega).

### ELISA

The amount of mouse IL-2 was assessed by mouse IL-2 Quantikine ELISA kit, according to the manufacturer's instruction (M2000, R&D systems, Minneapolis, MN).

### Immunohistochemistry

Acetone-fixed cryosections (7 μm) were blocked by immunoblock (DS Pharma Biomedical, Osaka, Japan) and then stained with the indicated Abs. The dilution of each antibody is shown in Supplementary Table 1. Fluorescent images of the sections were captured on a confocal laser scanning system (LSM-780, Carl Zeiss Microscopy GmbH, Göttingen, Germany). GL7^+^ area in the field was calculated using ImageJ software.

### Detection of anti-dsDNA antibody

Anti-dsDNA Ab in serum sample was detected by ELISA using double-stranded deoxyribonucleic acid coated plate and horse radish peroxidase conjugated goat anti-mouse IgG (H+L) antibody (1/5,000 diluted, Jackson Immuno Research, West Grove, PA).

### Detection of proteinuria

Uropaper III strip (Eiken Chemical Co., Tochigi, Japan) was used for urinalysis. Proteinuria was defined as urinary protein excretion exceeding 100 mg dl^−1^.

### Statistical analysis

Two sets of data were compared using two-tailed Student's *t*-test. When variances were not comparable, Welch's correction was applied. Sample sizes were selected based on the number of replicates (up to three). For survival rate or cumulative incidence, statistical analysis was performed by Log-rank test or Grey test with R software (http://www.R-project.org). *P* values <0.05 were considered statistically significant. Asterisk or NS means ‘significant' or ‘not significant', respectively. Each *in vitro* experiment was performed at least twice with reproducible results.

### Data availability

The authors declare that the data supporting of the findings of this study are available within the article and its Supplementary Information Files, or from the corresponding authors on reasonable request.

## Additional information

**How to cite this article:** Fujikura, D. *et al*. Death receptor 6 contributes to autoimmunity in lupus-prone mice. *Nat. Commun.* **8,** 13957 doi: 10.1038/ncomms13957 (2017).

**Publisher's note:** Springer Nature remains neutral with regard to jurisdictional claims in published maps and institutional affiliations.

## Supplementary Material

Supplementary Information Supplementary Figures, Supplementary Tables, Supplementary Methods and Supplementary References
