# Supplementary material for: Death receptor 6 contributes to autoimmunity in lupus-prone mice
Source: Nat Commun. 2017 Jan 3;8:13957. doi: 10.1038/ncomms13957 (PMC5216082; doi:10.1038/ncomms13957)
Supplement: Supplementary Information — Supplementary Figures, Supplementary Tables, Supplementary Methods and Supplementary References [file ncomms13957-s1.pdf]

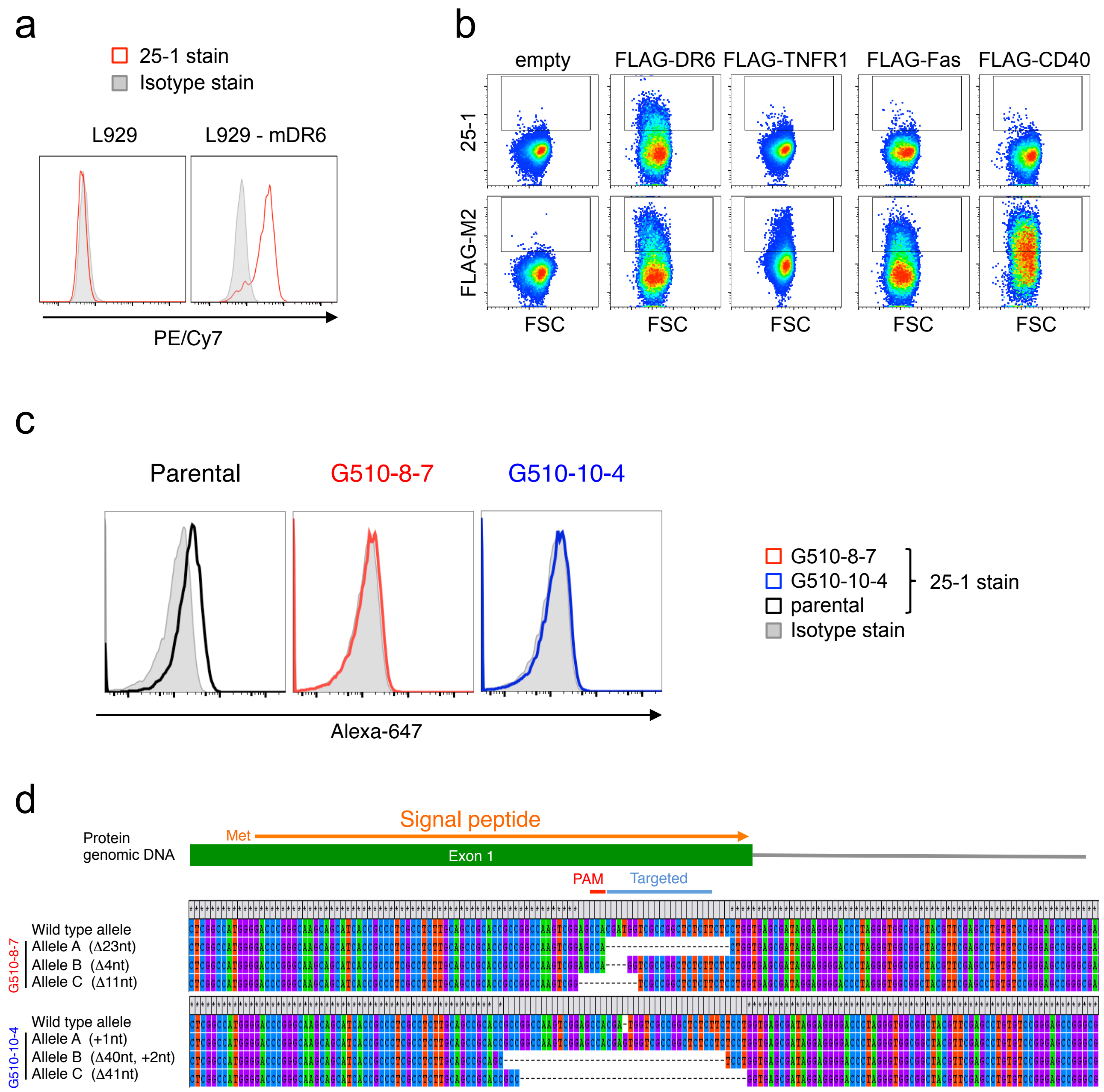

Supplementary Figure 1. Specificity of 25-1 Ab

(**a**) Parental L929 or L929 stably expressing mouse DR6 (L929-mDR6) was stained with 25-1Ab or isotype matched control plus PE/Cy7-conjugated secondary antibody against rat IgG. PE/Cy7 fluorescent signal of these cells was analyzed by flow cytometry. (**b**) HEK293T cells expressing the indicated gene, were also stained with 25-1Ab (upper panels) or anti-FLAG Ab (clone-M2, lower panels). The fluorescent signal of the cells was also analyzed. (**c**) Parental or DR6 deficient DO11.10-T cells (clone G510-8-7 and clone G510-10-4) were stained with Alexa fluor 647-conjugated 25-1 Ab or isotype matched control. Antibody specific signal of the cells was analyzed by flow cytometry. (**d**) Genomic DNA sequencing confirmed frame shift mutations on *Tnfrsf21* alleles of G510-8-7 or G510-10-4 single cell clone that were treated with the gene specific CRISPR-Cas9 as described in *Methods*. Genomic structure of exon1 of murine *Tnfrsf21* gene was schematically shown in upper. Gene sequence of *Tnfrsf21* gene allele of the indicated cell clone was shown in lower. Targeted region by the guide RNA was shown. Proto-spacer Adjacent Motif (PAM) was also shown.

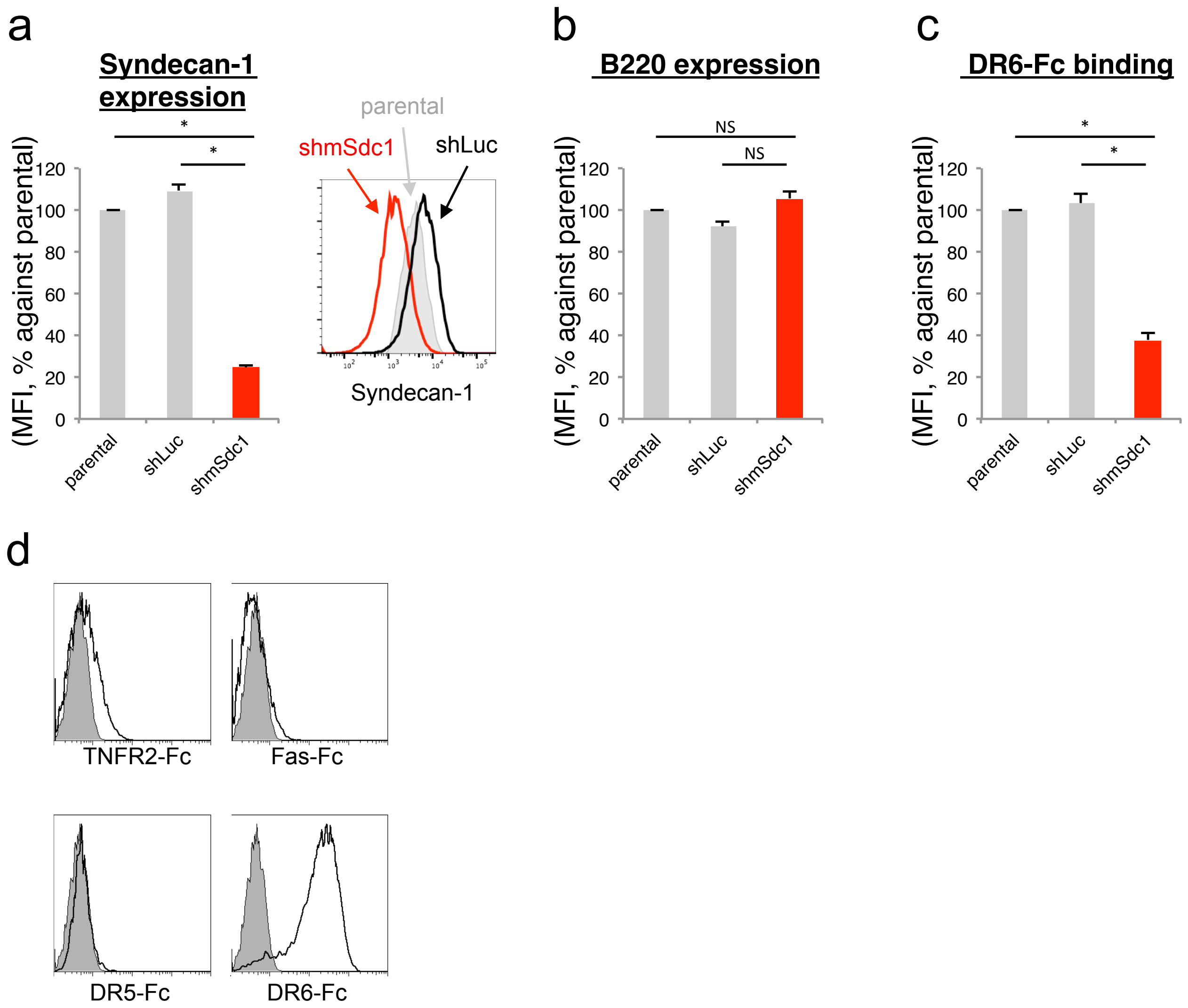

Supplementary Figure 2. Specific binding of syndecan-1 to DR6 ectodomain
Murine A20 cells were retrovirally transfected with plasmids encoding shRNA against *luciferase* (shLuc) or *Syndecan-1* (shmSdc1). (**a**) Endogenous syndecan-1 expression of the cells was analyzed by flow cytometry using anti-syndecan-1 Ab. Anti-syndecan-1 specific fluorescent signal of the indicated cells was shown (median of fluorescent intensity, MFI)(left). Error bar: SD (n = 3 per group). Asterisks indicate statistically significance ($p < 0.05$, t-test). A representative flow plot was also shown (right). (**b**) Non-specific effect of the shRNA treatment was also analyzed by flow cytometry using anti-B220 Ab. NS means not significant (n = 3 per group). (**c**) The indicated A20 cells were stained with DR6-Fc as described in Fig. 3**a** and then DR6-Fc specific fluorescent signal of the cells was detected by flow cytometry. Error bar: SD (n = 3 per group). Asterisks indicate statistically significance ($p< 0.05$, Student's t-test). (**d**) A20 cells, which endogenously express syndecan-1, was stained with Fc protein fused with ectodomain of the indicated TNFRSF molecules as described in **c**. Specific signal of the indicated recombinant protein of the cells was shown. Opened histogram means the indicated Fc fused protein stained. Closed histogram means control human IgG stained.

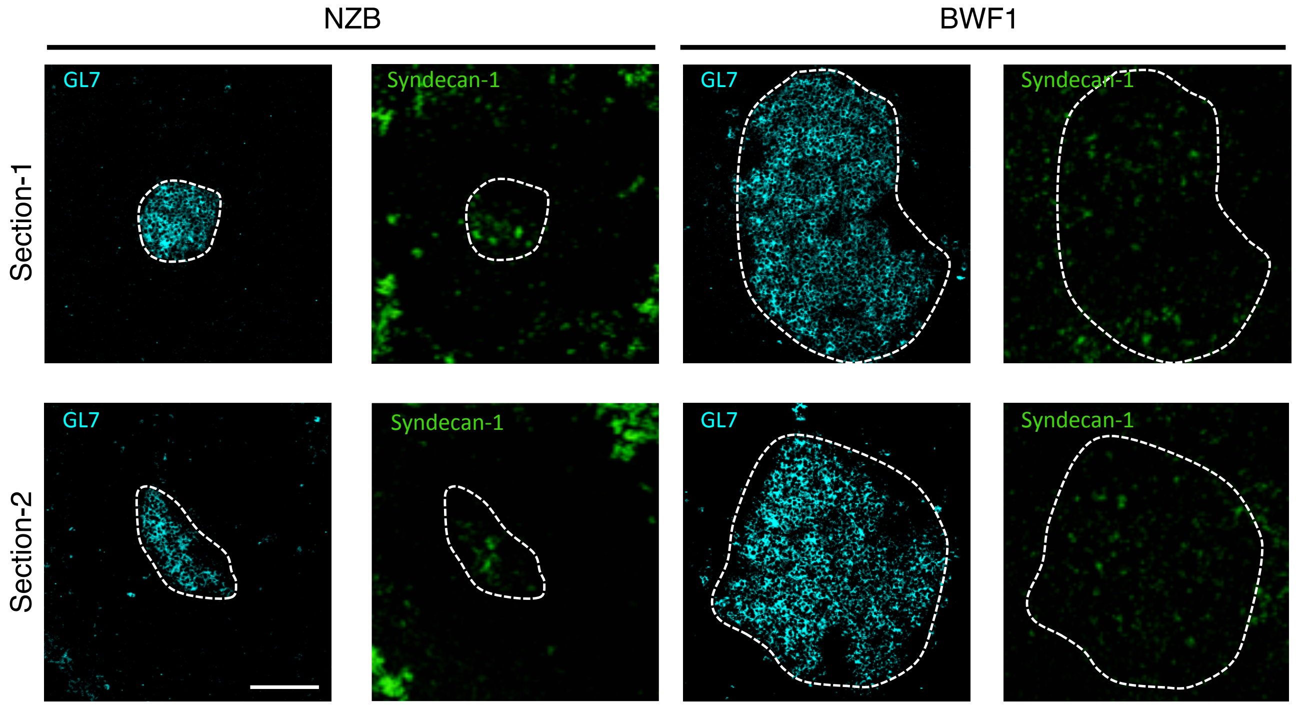

Supplementary Figure 3. Syndecan-1 expression of GC B cell in lupus-prone mice strains
Splenic cryosection obtained from 24-week-old female NZB or BWF1 mice were analyzed as described in Fig4**a** and **b**. Bar = 100μm

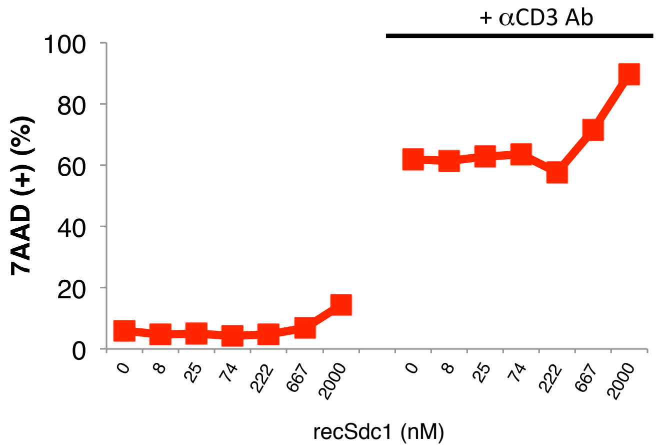

Supplementary Figure 4. Effect of recombinant syndecan-1 stimulation on cell death induction of DO11.10-T cells

DO11.10-T cells were stimulated with the indicated amount of recombinant syndecan-1 (recSdc1) with or without anti-CD3 Ab (αCD3 Ab, 10 μg ml^{-1}, immobilized). Seventeen hours after the stimulation, percentage of dead cell in each sample was assessed by 7AAD staining.

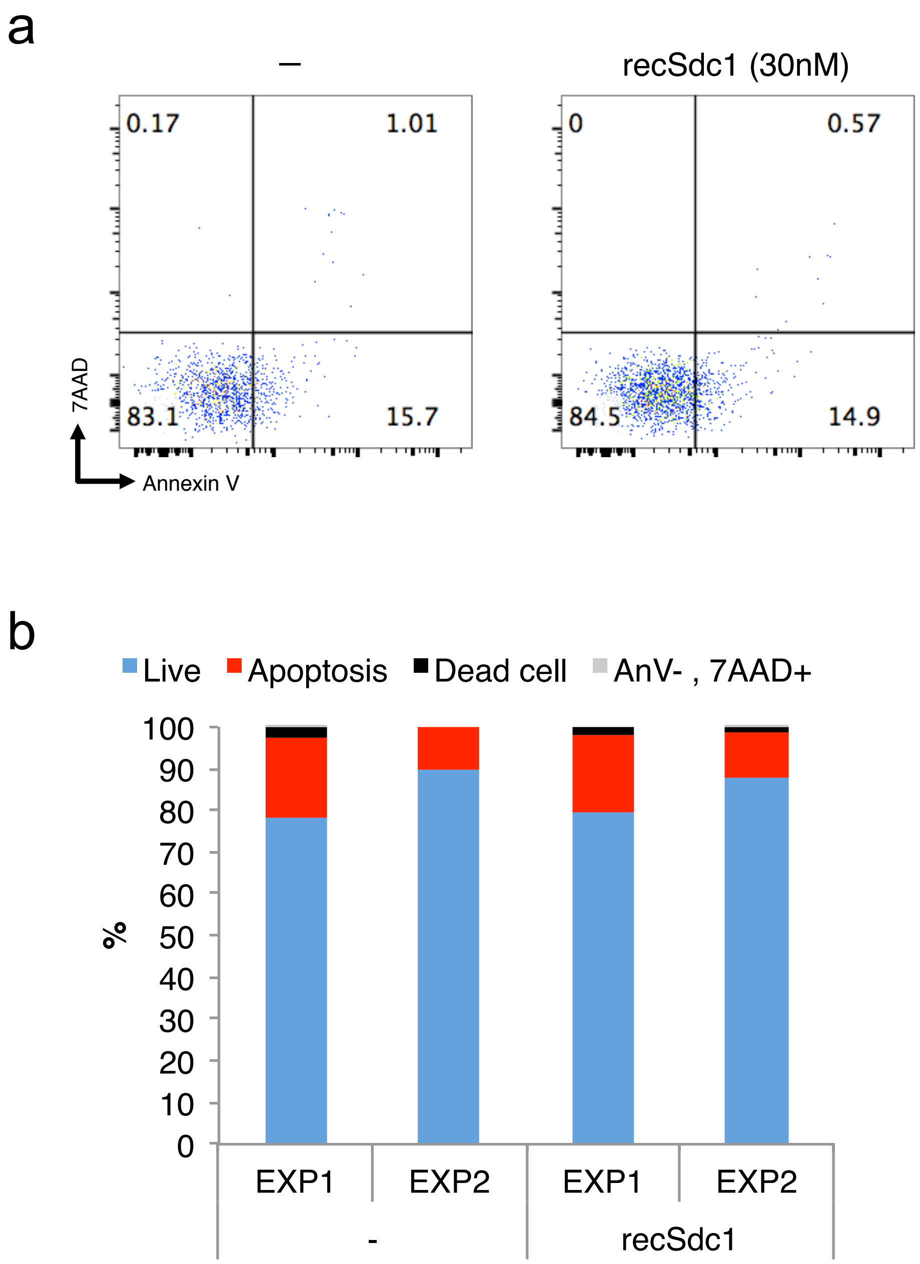

Supplementary Figure 5. Effect of recombinant syndecan-1 stimulation on apoptotic induction of isolated Tfh cells from lupus-prone mice

(**a**) $CXCR4^{+}$ $CXCR5^{high/int}$ $PD1^{high/int}$ $CD4^{+}$ cells were sorted from splenocytes obtained from 24-week-old female BWF1 mice. The cells were stimulated with or without recSdc1 (30 nM) under anti-CD3 Ab (10 μg ml^{-1}, immobilized) and anti-CD28 Ab (10 μg ml^{-1}, soluble) stimulations. Seventy-two hours after stimulation, apoptotic induction of lymphocyte-gated cells was assessed by Annexin V and 7AAD stains. These assays were carried out in two independent experiments. Annexin V^{-} $7AAD^{-}$, Annexin V^{+} $7AAD^{-}$ or Annexin V^{+} $7AAD^{+}$ cells were considered as live, apoptotic or dead cell, respectively. (**b**) Percentages of live, apoptotic or dead cells in lymphocyte gate were also shown in bar graph.

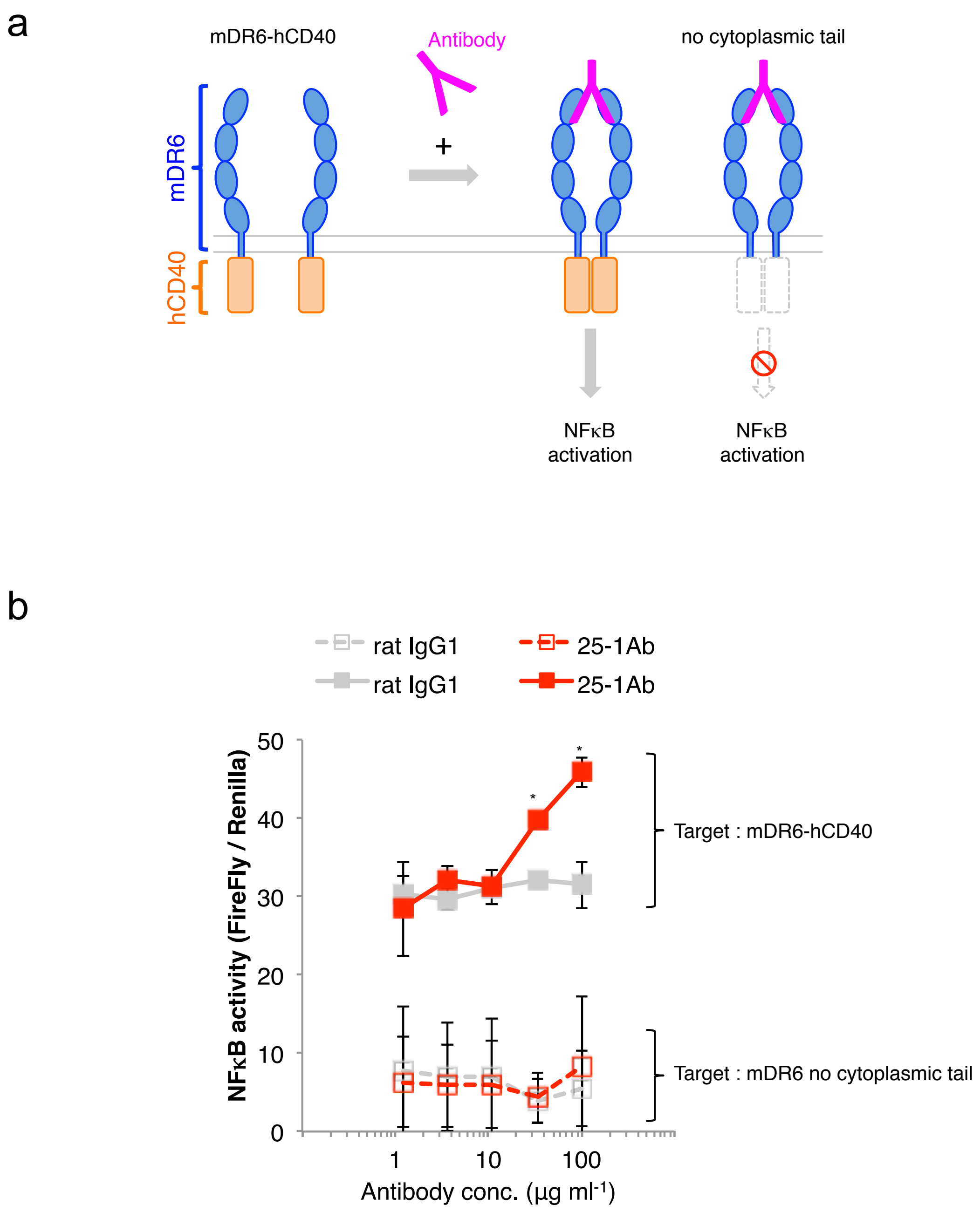

Supplementary Figure 6. DR6 receptor cross-linking mediated by 25-1Ab

(**a**) A scheme for DR6 receptor cross-linking was shown. The chimeric protein (mDR6-hCD40) consisting of both extracellular and transmembrane regions of murine DR6 (mDR6) and the cytplasmic tail of human CD40 (hCD40) was expressed in human embrionic kidney (HEK) cell line, 293T cells. Stimulation by antibody that has cross-linking activity for DR6, should activate well-characterized human CD40-dependent intracellular signal, which activates NFκB. The mDR6 mutant without cytoplasmic tail was used as negative control. (**b**) HEK293T cells were transfected with expression plasmid for mDR6-hCD40 or mDR6 without cytoplasmic tail, with NFκB-dependent reporter and reference plasmid as described in *Supplementary Methods*. Twenty-four hours after the transfection, the cells were stimulated with the indicated amount of the antibody. Seventeen hours after the stimulation, activities of luciferases of the cells were assessed as described in above. NFκB-dependent firefly luciferase activity was shown after normalized by renilla luciferase activity in each sample. Asterisks mean statistically significance compared with isotype matched control Ab ($p < 0.05$, Student's t-test). Error bar: SD (n = 3 per group).

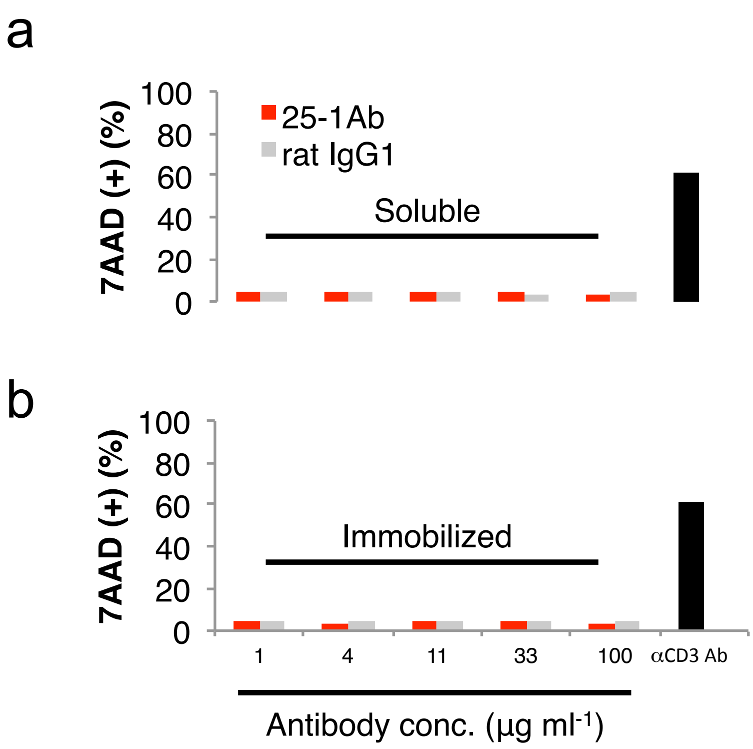

Supplementary Figure 7. Effect of 25-1 Ab on cell death induction in DO11.10-T cell

DO11.10-T cells were stimulated with the indicated amount of antibody (as soluble in **a** and immobilized in **b**). Seventeen hours after the stimulations, cell death induction in the cells was assessed by 7AAD stain. Anti-CD3 Ab (10 μg ml^{-1}, immobilized) was also used as positive control.

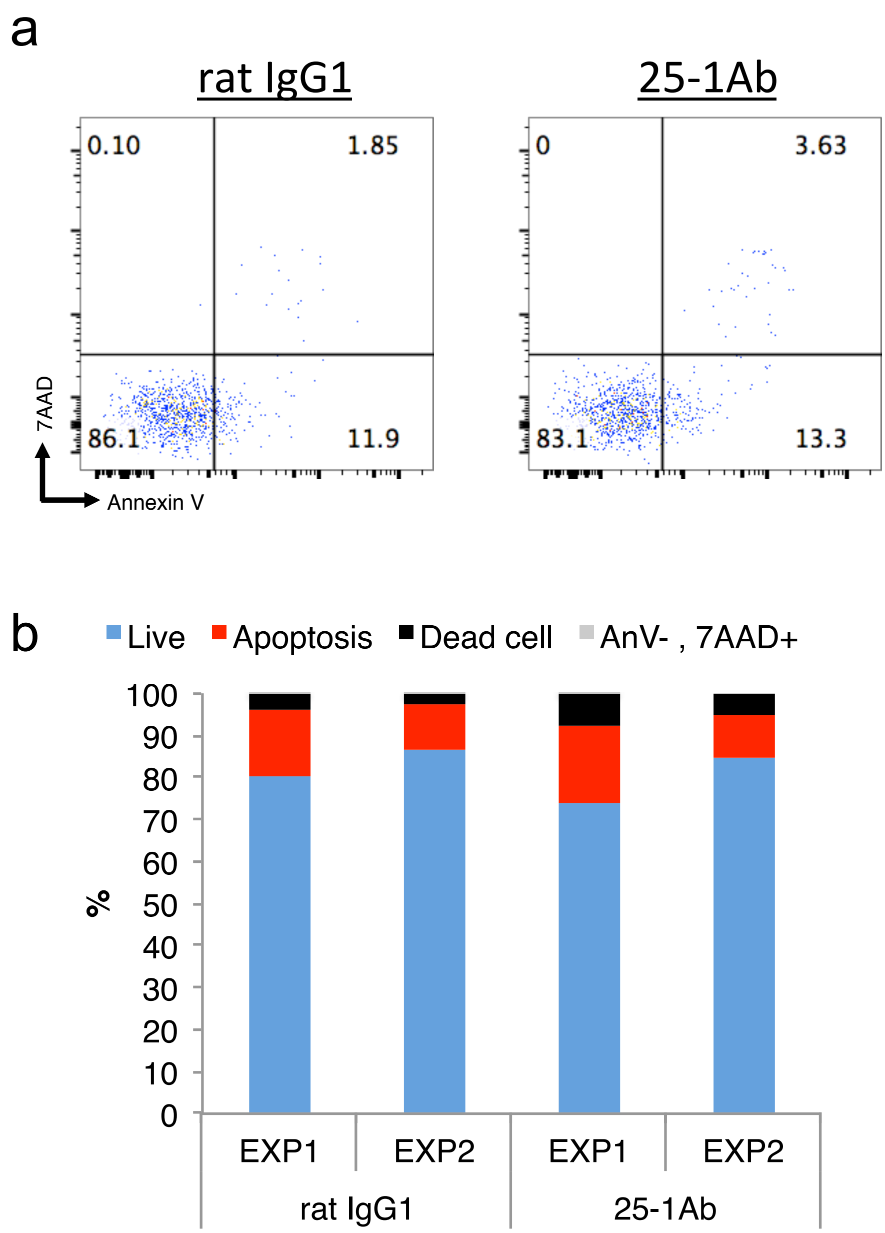

Supplementary Figure 8. Effect of 25-1Ab on cell death induction of Tfh cells of lupus-prone mice

(**a**) CXCR4^{+} CXCR5$^{high/int}$ PD1$^{high/int}$ CD4^{+} splenocytes isolated from 24-week-old female BWF1 were stimulated with 25-1Ab or its isotype matched Ab (10 µg ml^{-1}). Seventeen hours after the stimulations, apoptotic induction of the cells was assessed as described in Supplementary Fig. 5. Annexin V^{-} 7AAD^{-}, Annexin V^{+} 7AAD^{-} or Annexin V^{+} 7AAD^{+} cells were considered as live, apoptotic or dead cell, respectively. (**b**) These assays were carried out in two independent experiments. Percentages of live, apoptotic or dead cells in lymphocyte gate were also shown in bar graph.

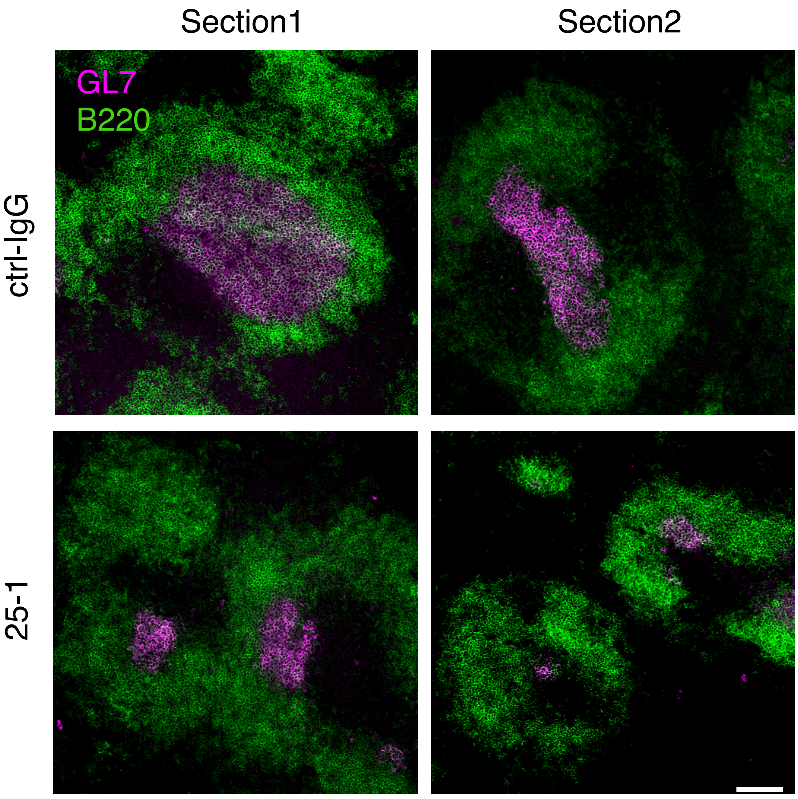

Supplementary Figure 9. Effect of 25-1Ab administration on splenic germinal center formation of lupus-prone mice

Splenic cryosection obtained from control rat IgG (ctrl-IgG)- or 25-1Ab (25-1)-treated female BWF1 mice was analyzed as described in Fig.5**h**. Bar = 100μm.

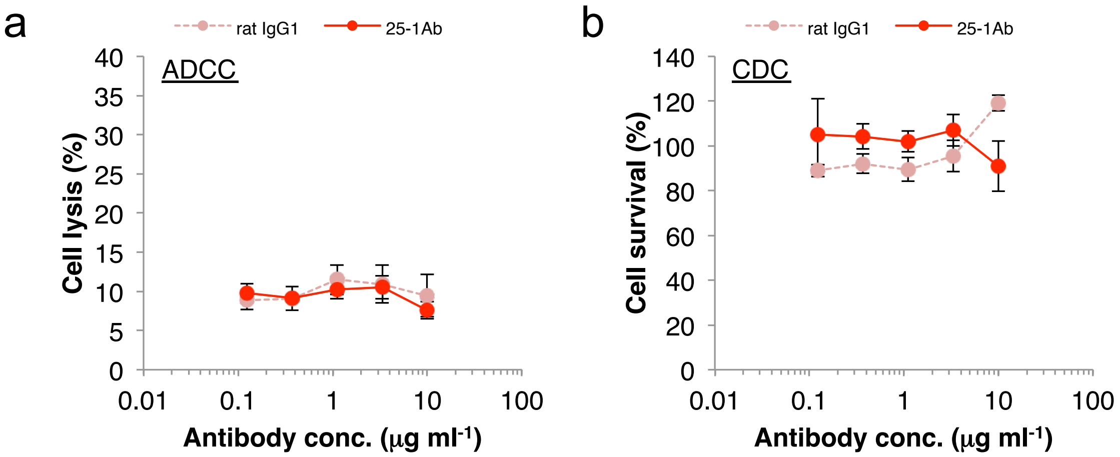

Supplementary Figure 10. Potential of 25-1Ab for ADCC and CDC

A potential of 25-1Ab for inducing ADCC (**a**) or CDC (**b**) was assessed as described in *Supplementary Methods*. Isotype matched rat IgG1 was used as control. Error bar: SD (n = 3 per group).

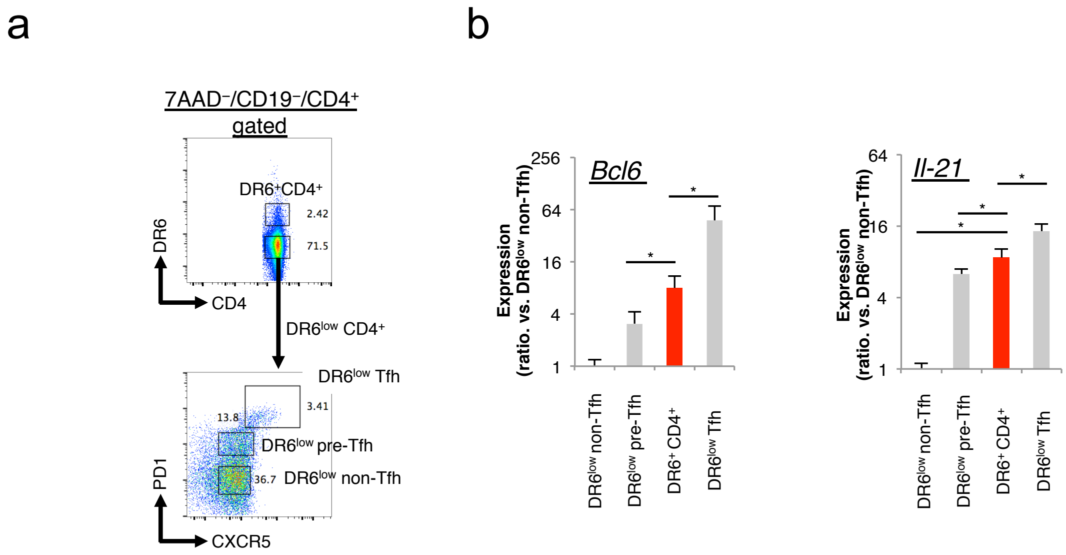

Supplementary Figure 11. Bcl6 and Il-21 expressions in DR6$^+$ CD4$^+$ cells of lupus-prone mice

(**a**) DR6$^+$ CD4$^+$ cells were sorted from 7AAD$^-$ CD19$^-$ CD4$^+$ splenocytes of 24-week-old female BWF1 mice. DR6^{low} Tfh, preTfh or nonTfh cells were also sorted from DR6^{low} CD4$^+$ splenocytes.
(**b**) *Bcl6* or *Il-21* gene expression of the cells was analyzed by qPCR. Error bar: SD (n = 3 per group). Asterisk means statistically significance ($p < 0.05$, Student's t-test).

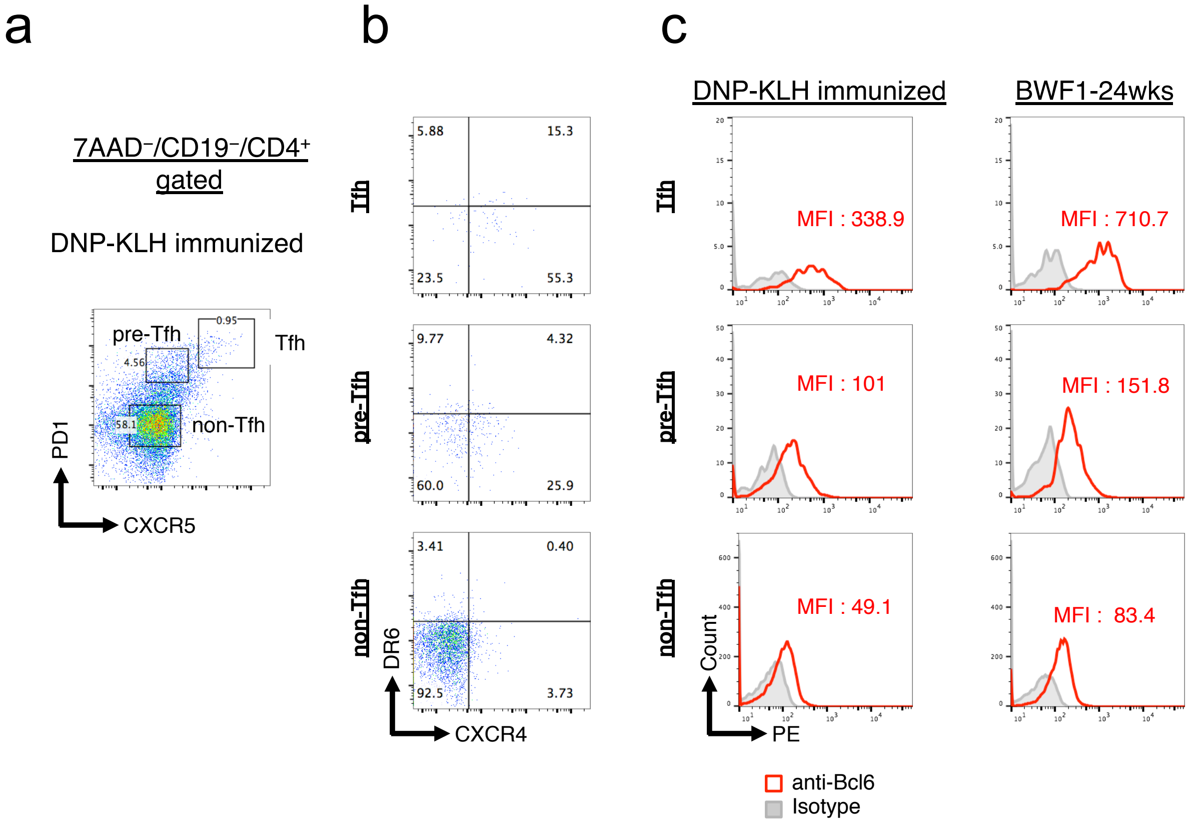

Supplementary Figure 12. DR6 expression on Tfh cells in DNP-KLH immunized B6 mice
Total splenocytes were obtained from B6 mice immunized with DNP-KLH at day 14 after the immunization. (**a**) The indicated Tfh cell populations were recognized as described in Fig.2**b**. (**b**) Expressions of DR6 and CXCR4 of the cells were analyzed as described in Fig.2**d**. (**c**) Bcl6 expression of Tfh, pre-Tfh or non-Tfh cells obtained from DNP-KLH immunized B6 (left panels) or 24-week-old female BWF1 mice (right panels) was analyzed as described in Fig.2**a**.

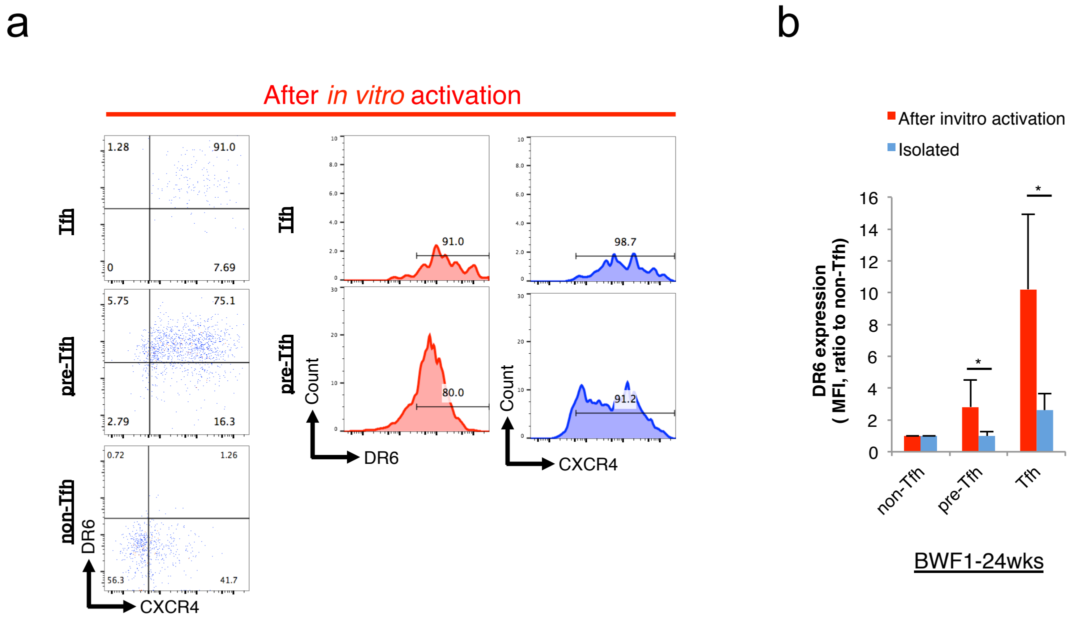

Supplementary Figure 13. DR6 expression on Tfh cells of lupus-prone mice during activation
(**a**) Total splenocytes isolated from 24-week-old female BWF1 mice were stimulated with anti-CD3 Ab (10 μg ml^{-1}, immobilized) plus anti-CD28 Ab (10 μg ml^{-1}, soluble). Three days after the stimulation, both DR6 and CXCR4 expression of the indicated cell populations were analyzed as described in Fig.2**d**. (**b**) Based on the data in **a** and Fig2**d**, anti-DR6 Ab specific signal (MFI, median) of the indicated cells was calculated. The results were shown as a ratio to means of non-Tfh in each experimental condition. Error bar: SD (n = 3 per group). Asterisk means statistically significance ($p < 0.05$, Student's t-test).

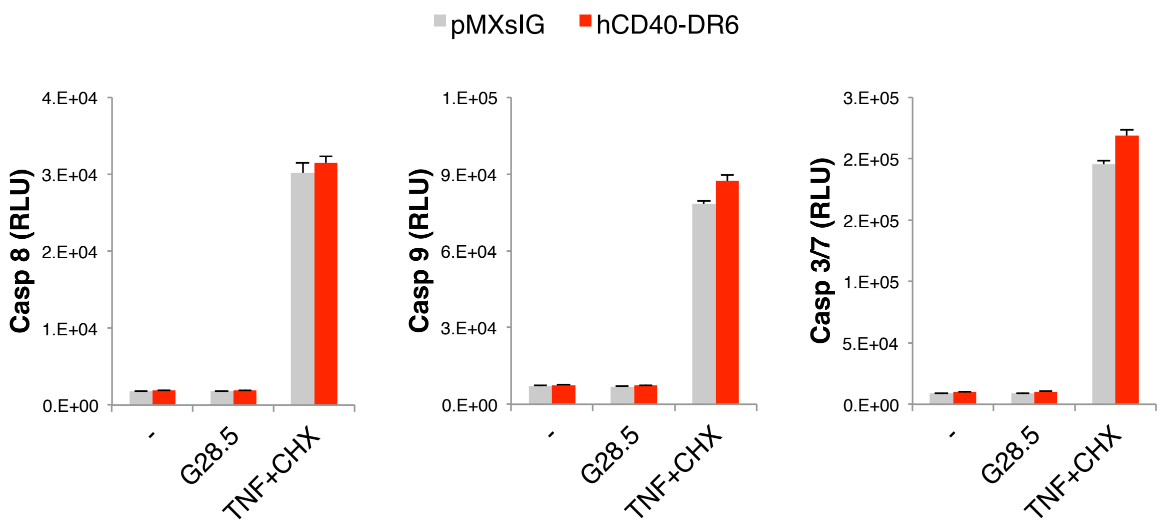

Supplementary Figure 14. Pro-apoptotic potential of DR6 cross-linking in DO11.10-T cells

Murine DO11.10-T cells stably transfected with empty pMXsIG or the plasmid for expressing hCD40-DR6 were stimulated with anti-human CD40 specific agonistic murine monoclonal antibody (clone G28.5, 10 μg ml^{-1}) or the combination of recombinant human TNFα (TNF, 20 ng ml^{-1}) plus cycloheximide (CHX,10 μg ml^{-1}). Three hours (for Caspase-8 or -9) or 4.5h (for Caspase 3/7) after the stimulation, the indicated caspase activities of the cells were assessed as described in *Supplementary Methods*. Error bar means standard deviation (n = 3 per group).

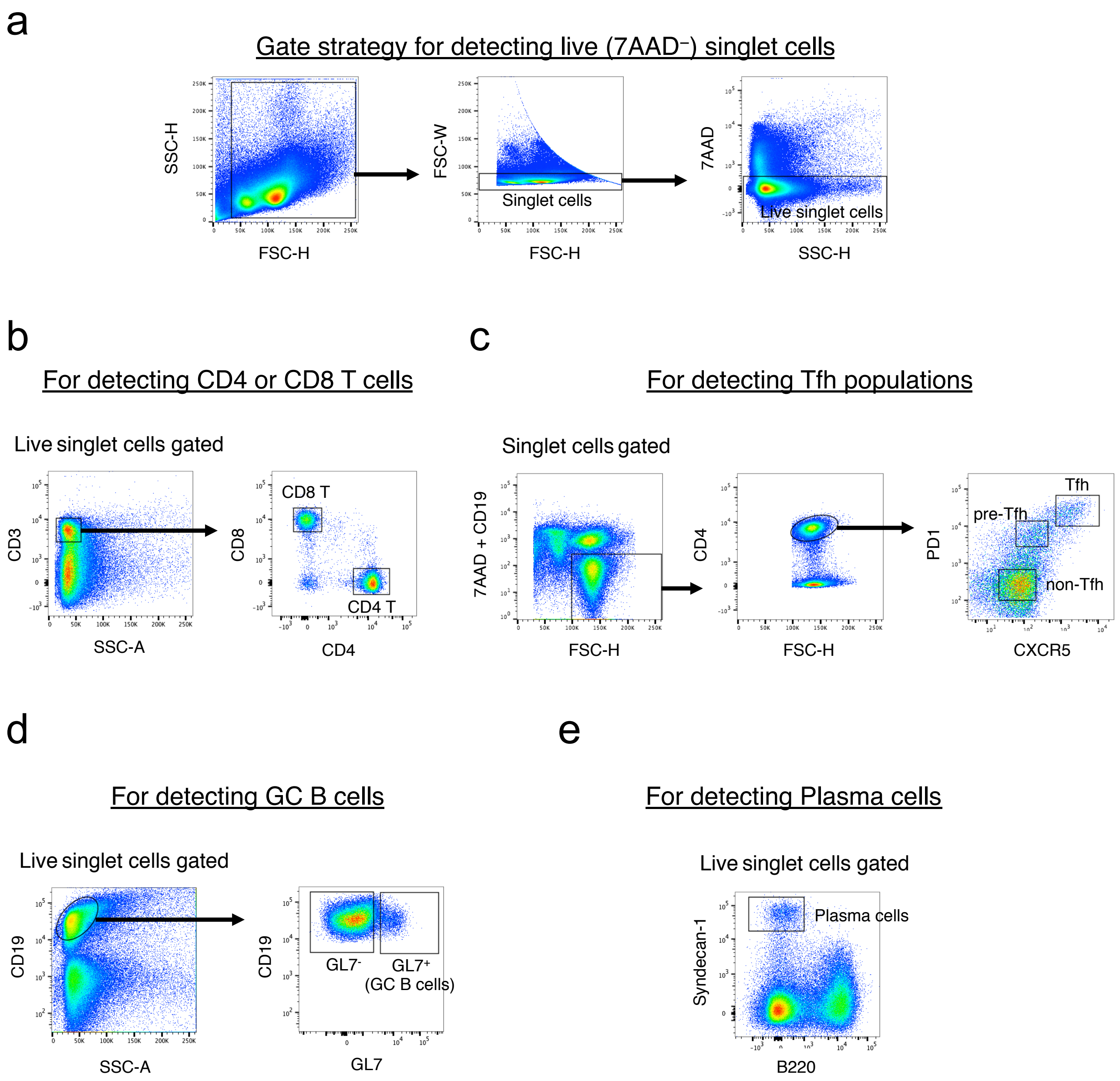

Supplementary Figure 15. Scheme of gating strategies that were used in the present study

Murine splenocytes that were stained as described in *Methods* were analyzed on flow cytometry. (**a**) Splenocytes among the stained sample were recognized on SSC-Height (H) vs. FSC-H dot plot. Among the splenocytes, singlet cells were also discriminated on FSC-H vs. FSC-Width (W) dot plot. Among the singlet cells, live singlet cells were detected as 7AAD^{-} cells on SSC-H vs. 7AAD dot plot. (**b**) For detecting CD4^{+}- or CD8^{+} T cells, the live singlet cells were separated on SSC-Area (A) vs. CD3 dot plot. CD3^{+} cells were further separated on CD4 vs. CD8 dot plot. (**c**) For analyzing Tfh cell populations, singlet cells were analyzed on FSC-H vs. 7AAD plus CD19. Fluorescent signal of PerCP/Cy5.5-conjugated anti-CD19 Ab was detected by 7AAD detector channel. Among the 7AAD^{-} CD19^{-} cells, CD4^{+} cells were detected on FSC-H vs. CD4 dot plot. On CXCR5 vs. PD1 dot plot, the CD4^{+} cells were further separated as CXCR5^{high} PD1^{high} Tfh, PD1^{int} CXCR5^{int} preTfh or CXCR5^{-} PD1^{-} nonTfh cells as shown. (**d**) For detecting GC B cells, live singlet cells were separated on SSC-A vs. CD19 dot plot. CD19^{+} cells were further separated on GL7 vs. CD19 dot plot. GL7^{-} or GL7^{+} cells were considered as non GC- or GC B cells, respectively. (**e**) For assessing frequency of plasma cells among total splenocytes, live singlet cells were analyzed on B220 vs. syndecan-1 dot plot. Syndecan-1^{high} B220^{-} cells were considered as plasma cells.

Supplementary Table 1. List of antibodies used in the present study			
**Antigen**	**clone name**	**concentration**	**Company**
CD3	145-2C11	1/50 dilution (for staining)	Biolegend (San Diego, CA)
CD4	YTS191.1	1/10 dilution	Beckman Coulter (Brea, CA)
CD8	KT15	1/10 dilution	Beckman Coulter
PD1	RMP1-30	1/80 dilution	Biolegend
CXCR5	L138D7	1/40 dilution	Biolegend
CXCR4	2B11	1/200 dilution	BD Pharmingen (San Diego, CA)
B220	RA3-6B2	1/50 dilution	Biolegend
CD11c	N418	1/200 dilution	Biolegend
CD49b	DX5	1/50 dilution	BD Pharmingen
F4/80	BM8	1/80 dilution	Biolegend
Ly6G	1A8	1/200 dilution	Biolegend
CD19	1D3	1/80 dilution	BD Pharmingen
syndecan-1	281-2	1/20 dilution	Biolegend
GL7	GL7	1/50 dilution	Biolegend
CD28	37.51	10 µg/ml (for in vitro stimulation)	BD Pharmingen
CD16/32	2.4G2	20 µg/ml	BD Pharmingen
FLAG	M2	10 µg/ml	Sigma Aldrich (St. Louis, MO)
Bcl6	K112-91	1/50 dilution	BD Pharmingen

Supplementary Table 1. List of antibodies used in the present paper

Supplementary Table 2. List of sequences for primers used in the present study		
**Targeted gene**	**Forward primer**	**Reverse primer**
Tnfrsf21	5'-GCAACGGCCATGGTATTGAC-3'	5'-TATCCATTGGAGAAGGCCGC-3'
Il-21	5'-CACATAGCTAAATGCCCTTCCTG-3'	5'-GGTACCCGGACACAACATGG-3'
Bcl6	5'-AGTTTCTAGGAAAGGCCGGACAC-3'	5'-CCATGTTGTGTTTGCCCAGTG-3'
Actb	5'-CACTGTCGAGTCGCGTCC-3'	5'-TCATCCATGGCGAACTGGTG-3'

Supplementary Table 2. List of sequences for primers used in the present paper

Supplementary Methods

Plasmids

The expression plasmids for FLAG-TNFR1, Fas or DR6 were also used[1]. For the plasmid expressing FLAG-CD40, cDNA encoding CD40 without signal sequence was cloned in frame into the expression plasmid pFLAG-CMV-1 (Sigma Aldrich, St. Louis, MO). The expression plasmids for TNFR2-Fc, Fas-Fc and DR5-Fc were also constructed by inserting cDNA fragment encoding mouse TNFR2(+1_+259), mouse Fas(+1_+169) or mouse DR5(+1 _+177) into pcDNA3 as described above. For murine *Syndecan-1* specific shRNA production, synthetic DNA fragment encoding small hairpin RNA targeting on mRNA sequence of murine *Syndecan-1* (5'-GAGGTCTACTTTAGACAACTT-3')[2] was inserted into pSIREN-RetroQ-DsRed (Clontech, Palo Alto, CA).

ADCC and CDC

Antibody-Dependent-Cellular-Cytotoxicity (ADCC) was evaluated using a LDH release assay. L929 cells stably expressing both ectodomain and transmembrane region of murine DR6 (L929-mDR6), as target, were seeded (1×10^4 cells per well) in Dulbecco's modified Eagle's medium (DMEM) containing 2% BSA on round bottom 96-well plates. The indicated amount of antibody and human peripheral mononuclear cells as effector were added into the same well (Target : Effector ratio is 1 : 20). Six hours after incubation, activity of LDH released in supernatant was assessed by using Cytotoxicity Detection Kit^{PLUS} (LDH) (Roche). Percent of cytotoxicity was calculated from the following formula: percent specific lysis = (LDH activity in test sample − in non effector nor antibody sample) / (Maximum release − in non effector nor antibody). Complement dependent cytotoxicity (CDC) was determined by WST assay. L929-mDR6 cells as target cells (1×10^4 cells per well) were incubated in DMEM containing 20% non inactivated rabbit serum as a source of complement and the indicated amount of antibody for 6 h. Viability of target cells was determined by WST assay-based Cell counting kit-8 (Dojindo Laboratories, Kumamoto, Japan). Percent of survival was calculated as percentage of means obtained from no-antibody group.

Induction of DR6 signal by using chimeric protein

For expressing hCD40-DR6[1], cDNA encoding extracellular and transmembrane region of human CD40 was joined with cDNA encoding cytoplasmic tail of DR6. The resulted cDNA was cloned into pMXsIG. The resulted or empty plasmid was retrovirally transfected into DO11.10-T cell hybridoma. enhanced green fluorescent protein positive transfectants were sorted as described above. The transfectants were seeded on white-wall 96 well plate (2×10^4 cells per well) and stimulated with anti-human CD40 specific agonistic murine monoclonal antibody (clone G28.5, 10 μg ml^{-1}). The stimulation with recombinant human TNFα (20 ng ml^{-1}) with cycloheximide (CHX,10 μg ml^{-1}) was also used as positive control. Three hours (for Caspase8) or 4.5h (for Caspase-3/7 and Caspase9) after the stimulation, the activity of the indicated caspase in the cells was assessed by using Caspase Glo assay reagent according to the manufacturer's instructions (Promega).

Assessing DR6-cross-linking activity of antibody

Plasmid for expressing mDR6-hCD40 was constructed as described above. HEK293T cells were transiently transfected in 12-well tissue culture plate using Polyethylenimine "Max" (Polysciences, Inc., Warrington, PA) with the plasmid for mDR6-hCD40 or mDR6 without cytoplasmic tail (3 μg per well) together with firefly luciferase reporter plasmid containing 5 × κB binding site (3 μg per well). The plasmid phRG-TK (0.5 μg per well) was used as internal control. Twenty-four hours after transfection, cells were suspended and reseeded (2×10^4 cells per well) on white-wall 96-well plate that was coated with the indicated amount of the antibody. Seventeen hours after the incubation, the activities of firefly and renilla luciferase of the cells were detected as described in above.

Detection of apoptosis

Cells were twice washed with ice-cold PBS and then stained in the buffer containing 10 mM HEPES (pH7.4), 2.5 mM $CaCl_2$, 140 mM NaCl, APC-conjugated Annexin V (1/20 dilution, BD Biosciences) and 7AAD (1/100 dilution, Biolegend) at room temperature in the dark. Fifteen minutes after staining, cells were analyzed by flow cytometry. Annexin V^- $7AAD^-$, Annexin V^+ $7AAD^-$, Annexin V^+ $7AAD^+$ cells in lymphocyte gate were considered as live, apoptotic or dead cells, respectively.

Supplementary References

1. Fujikura, D. *et al*. CLIPR-59 regulates TNF-alpha-induced apoptosis by controlling ubiquitination of RIP1. *Cell Death & Disease* **3** (2012).

2. McQuade, K.J., Beauvais, D.M., Burbach, B.J. & Rapraeger, A.C. Syndecan-1 regulates alpha(v)beta(5) integrin activity in B82L fibroblasts. *Journal of Cell Science* **119**, 2445-2456 (2006).
